# Unraveling the Genetic Basis of Key Agronomic Traits of Wrinkled Vining Pea (*Pisum sativum* L.) for Sustainable Production

**DOI:** 10.3389/fpls.2022.844450

**Published:** 2022-03-14

**Authors:** Admas Alemu, Agnese Kolodinska Brantestam, Aakash Chawade

**Affiliations:** ^1^Department of Plant Breeding, Swedish University of Agricultural Sciences, Alnarp, Sweden; ^2^Nomad Foods Ltd., Findus Sverige AB, Bjuv, Sweden

**Keywords:** wrinkled vining pea yield, downy mildew, DArTseq, GWAS, GBS

## Abstract

Estimating the allelic variation and exploring the genetic basis of quantitatively inherited complex traits are the two foremost breeding scenarios for sustainable crop production. The current study utilized 188 wrinkled vining pea genotypes comprising historical varieties and breeding lines to evaluate the existing genetic diversity and to detect molecular markers associated with traits relevant to vining pea production, such as wrinkled vining pea yield (YTM100), plant height (PH), earliness (ERL), adult plant resistance to downy mildew (DM), pod length (PDL), numbers of pods per plant (PDP), number of peas per pod (PPD), and percent of small wrinkled vining peas (PSP). Marker-trait associations (MTAs) were conducted using 6902 quality single nucleotide polymorphism (SNP) markers generated from the diversity arrays technology sequencing (DArTseq) and Genotyping-by-sequencing (GBS) sequencing methods. The best linear unbiased prediction (BLUP) values were estimated from the two-decades-long (1999–2020) unbalanced phenotypic data sets recorded from two private breeding programs, the Findus and the Birds eye, now owned by Nomad Foods. Analysis of variance revealed a highly significant variation between genotypes and genotype-by-environment interactions for the ten traits. The genetic diversity and population structure analyses estimated an intermediate level of genetic variation with two optimal sub-groups within the current panel. A total of 48 significant (*P* < 0.0001) MTAs were identified for eight different traits, including five for wrinkled vining pea yield on chr2LG1, chr4LG4, chr7LG7, and scaffolds (two), and six for adult plant resistance to downy mildew on chr1LG6, chr3LG5 (two), chr6LG2, and chr7LG7 (two). We reported several novel MTAs for different crucial traits with agronomic importance in wrinkled vining pea production for the first time, and these candidate markers could be easily validated and integrated into the active breeding programs for marker-assisted selection.

## Introduction

Pea (*Pisum sativum* L., 2*n* = 14) is the second most essential legume crop next to common bean globally with an annual production of 14.2 million tons of dry pea and 21.6 million tons of vining pea in 2019^1^. Wrinkled vining pea for the freezing industry is harvested as immature pea seeds (green peas) and consumed as a vegetable. Green peas have a nutritionally favorable composition with respect to macronutrients (low-fat content, high in protein and fiber) and micronutrients (source for vitamins, antioxidants, and minerals, e.g., bioavailable iron) and have a low glycemic index contributing to the health benefits for consumers (Foster-Powell and Miller, 1995; Moore et al., 2018; National food administration, 2021). Furthermore, as a member of the legume family, pea shares the symbiosis feature of fixing the atmospheric nitrogen with the soil bacteria (*Rhizobacteria*) that makes the plant a good source of nitrogen fertilizer, thereby playing a role in alleviating the greenhouse gas emissions (Crews and Peoples, 2004; Nemecek et al., 2008).

Pea has been cultivated so long in the Fenno-Scandinavia countries, including Denmark, Finland, Norway, and Sweden since its introduction to the area around 6,000 BP (Hjelmqvist, 1979; Hagenblad et al., 2014). Sweden is notably home to a vast gene pool of different types of peas, and a large number of cultivars and landraces were grown widely in the 19th century. Several historical landraces and cultivars have been preserved and available in gene-banks, such as in the Nordic Genetic Resource Center (NordGen) in Sweden and John Innes Centre in England (Hagenblad et al., 2014). Field/dry peas and vegetable peas are the two broad categories of pea types, and vegetable peas are further grouped into green/garden/shelling peas, snow peas, and snap/sugar peas (Myers et al., 2001). Currently, various types of peas are cultivated across the country, covering the areas from the southern tip of Sweden to near the polar circle (Vanhala et al., 2016; Carlson-Nilsson et al., 2021). In addition, efforts have been made to adapt pea genotypes to new environments in the Arctic region (Carlson-Nilsson et al., 2021). Hence, several abiotic stresses are of key importance in the Nordic region including early spring frost, early spring drought, and late summer drought, which affects all agricultural crops grown in the region (Chawade et al., 2018).

Pea has been a model plant in genetics since the 18th century. The famous geneticist Gregor Mendel discovered the laws of genetics and inheritance, such as dominance, segregation, and independent assortment, using different garden pea morphotypes (Mendel, 1865). However, the genomics of pea has lagged, and its whole genome sequence has been discovered lately (Kreplak et al., 2019). Consequently, the availability of the pea reference genome paves the way to discover favorable alleles underlying the phenotypic variations of traits with agronomic importance and accelerate trait improvement *via* genomic tools such as marker-assisted selection (MAS) and genomic selection (GS). Estimating the existing genetic variation within particular germplasm is a critical component of plant genetics, breeding, and evolution (Peterson et al., 2014). This is because the characterization of the available genetic diversity and allelic distribution of germplasm is an essential component in conservation as well as a selection of parents for breeding with diverse genetic background (Upadhyaya et al., 2011). The single nucleotide polymorphism (SNP) markers are the most high-throughput and abundantly existing DNA markers in crop plants (Morgil et al., 2020). The emergence of various next-generation sequencing (NGS) technologies particularly allowed the SNPs as a primary tool to exploit the genetic resource of several crops (Kumar et al., 2012).

The GBS is a robust one-step method for simultaneous genotyping and SNP discovery (Elshire et al., 2011). The GBS alleviated the large, complex, and repeated sequences of plant genomes by reducing them *via* restriction enzymes (REs) digestion followed by high-throughput sequencing (Elshire et al., 2011; He et al., 2014). Subsequently, the SNP discovery with GBS has been successfully applied in several crops, including pea (Boutet et al., 2016; Gali et al., 2019; Dissanayaka et al., 2020). The DArT discovered the other method of high-throughput genotyping, which is combining GBS with the next-generation sequencing platforms called NGS-DArTseq, or simply DArTseq. This approach can generate SNP markers with higher numbers covering the plants’ whole genome (Akbari et al., 2006; Raman et al., 2014; Barilli et al., 2020) and is successfully used in several crops, including pea (Aznar-Fernández et al., 2020). The DArTseq method reduces the complexity of the genome through digestion with restriction enzymes followed by sequencing of short reads, predominantly corresponding to the active genes (Tomkowiak et al., 2021). The GBS and DArTseq genotyping platforms produce high-throughput and abundant SNP markers that have been widely utilized to estimate genetic variation, discover causative allelic variations, and understand the contributing genetic architecture of complex traits with economic importance (Baloch et al., 2017).

A genome-wide association study (GWAS) has been widely used for the last couple of decades as a primary tool in detecting the genetic background of polygenic traits, and this technique has been converted from a promising new tool to a robust, ubiquitous technique for understanding complex traits in various crops (Tibbs Cortes et al., 2021). Independently or along with bi-parental linkage-based quantitative trait locus (QTL) mapping, the GWAS has been successfully utilized to detect numerous traits with economic importance in pea, including abiotic stress (Klein et al., 2014; Beji et al., 2020; Tafesse et al., 2020), biotic stress (Desgroux et al., 2016, 2018; Aznar-Fernández et al., 2020), yield and yield-related traits (Gali et al., 2019; Klein et al., 2020), and quality-related traits (Dissanayaka et al., 2020).

Breeding programs test large sets of inbred lines in multiple environments for extended years. These recorded phenotypic data sets from the breeding materials are commonly a rich source of allelic variations that could be used to discover marker-trait associations with economically relevant traits. However, the breeding-program-derived data sets are generally unbalanced since excluding some of the underperformed lines and adding new lines are parts of the process throughout the breeding cycle, making the dataset intricate (Bernardo, 2008). Nonetheless, the availability of mixed models that integrates the years and environments followed by estimating the mean genetic effects of individual inbred lines, such as best linear unbiased prediction (Smith et al., 2005), made it possible to utilize such data sets in the marker-trait discovery *via* the GWAS analysis. As a result, the historically recorded unbalanced phenotypic datasets found in breeding programs have been successfully used to detect relevant QTLs and markers in different crops associated with traits such as yield, quality, and disease resistance (Kraakman et al., 2004; Pozniak et al., 2012; Wang et al., 2012; Fiedler et al., 2017; Johnson et al., 2019; Liu et al., 2019; Thompson et al., 2021). In addition, the GS accuracy greatly improved when the models trained with populations comprised historical phenotypic datasets in breeding programs (Dawson et al., 2013; Gapare et al., 2018). The prediction accuracy is further leveraged when the SNP markers identified in GWAS results are fitted as fixed effects (Odilbekov et al., 2019; Alemu et al., 2021).

This study exploited 188 wrinkled vining pea genotypes encompassing varieties and breeding lines developed from Nomad Foods’ breeding programs with the following objectives: (I) to estimate the existing genetic variation, population structure, and linkage disequilibrium, and (II) to detect the SNP markers associated with economically relevant traits related to wrinkled vining pea yield and downy mildew resistance. To accomplish this, the best linear unbiased predictions were estimated from the breeding programs derived two-decades-long historical unbalanced data sets from 1999 to 2020, for ten different traits. Genotyping of the current wrinkled vining pea panel was done with both DArTseq and GBS sequencing methods.

## Materials and Methods

### Plant Material

A total of 188 wrinkled vining pea varieties and lines were assembled for this study (Supplementary Table 1). The panel comprises 179 genotypes from Findus (now part of Nomad Foods) in Sweden encompassing 164 breeding lines, seven out of 12 varieties are currently used in crop production, and five have been used historically and now delisted, as well as three reference lines: for downy mildew resistance (1 genotype) and susceptibility (1 genotype), and a source line for tolerance against *Aphanomyces euteiches* root rot (1 genotype). The other nine genotypes were originated from Birds Eye (now part of Nomad Foods) in the United Kingdom, which included six breeding lines and three varieties.

### Phenotypic Data and Experimental Design

The phenotypic data of 10 different agronomic traits related to wrinkled vining pea yield, ERL, and adult plant resistance to DM were extracted from the unbalanced historical data recorded for the past 2 decades (1999–2020) in Nomad Foods breeding programs. The genotypes were tested in various locations of southern Sweden, with a total of 192 year-by-location combinations (environments hereafter). The phenotypic data from these trails were included for the GWAS analysis. The number of trials in a year ranged from three in 2019 to eleven in 2001, with an average of eight locations per year. The number of genotypes per year varied in a range of 17 in 2007 to 77 in 2020, with an average of 30 genotypes. Individual genotypes were tested for a minimum of two environments per year to a maximum of 15 environments, with an average of seven environments. The trials were connected by the ten used checks across all experiments to account for the environmental effects, followed by mean adjustment including the widely known pea varieties, “Bikini” and “Avola.” The field trial experimental design was based on the incomplete block design with two replications. The following agronomic and disease resistance traits were included in the current GWAS analysis: Above-ground biomass (BM), PH, ERL, YTM100, PSP, number of pods per plant (PDP), number of pods per node (PDN), Pod length (PDL), number of peas per pod (PPD), and adult plant resistance to DM. The above-ground biomass (kg) was recorded from the total biomass of plants from the harvested area of 10 m^2^, excluding roots, while the plant height was taken from 10 plants/plot average values (cm). The ERL or relative maturity is expressed in days, earlier (−) or later (+) to the control variety “Cabree.” The yield (YTM100) was defined as the wrinkled vining pea yield at a 100-tenderometer value (t/ha). The percent of small wrinkled vining peas was calculated from the mass of small wrinkled vining peas divided by the total wrinkled vining pea yield. The small peas are those with less than 8.7 mm in diameter and are considered as the higher quality standard in wrinkled vining pea production. The number of pods with developed wrinkled vining peas per plant was calculated from 10 plants/plot aggregate values. The number of pods per node was counted from the aggregate value of the second node of 10 plants per plot. The pod length (mm) was measured from the plant’s second node, and the average score was taken from 10 plants per plot. The number of wrinkled vining peas per pod was measured from the second node and taken as the average values of 10 plants/plot. Adult plant resistance to DM was measured as the ratio of pods that were free of DM from the total number of infected pods per plant and were taken from the average of 10 plants/plot.

### DNA Extraction and Single Nucleotide Polymorphism Discovery by Diversity Arrays Technology Sequencing and Genotyping-by-Sequencing

For DNA extraction, the plants were grown in a growth chamber at Findus in Sweden for 19 days at 20°C. A pooled leaf tissue sample of four plants per line was freeze-dried and outsourced for DNA extraction and SNP genotyping with both DArTseq and GBS sequencing methods. Following the proprietary methodology, a high-throughput DArTseq SNP genotyping was performed at the Diversity Arrays Technology Pty. Ltd., Canberra, Australia^2^. Briefly, the DNA samples were subjected for complexity reduction by digestion/ligation reactions following the procedure by Kilian et al. (2012), but replacing the single *Pst*I-compatible adaptor with two adaptors corresponded for the two different restriction enzymes (*Pst*I and *Mse*I) overhangs. The *Pst*I-compatible adapter was designed to include Illumina flowcell attachment sequence, sequencing primer sequence, and barcode region, while the reverse adapter contained flowcell attachment region and *Mse*I-compatible sequence. The only “mixed fragments” (*Pst*I*-Mse*I) were effectively amplified in PCR for 30 rounds using the most optimal reaction conditions as follows: 94°C for 1 min (initial denaturation), 30 cycles each with 94°C for 20 s (denaturation), 58°C for 30 s (annealing), 72°C for 45 s for extension, and 72°C for 7 min final extension. Subsequently, each sample’s equimolar amounts of amplification products were bulked and sequenced with the HiSeq 2000 (Illumina^®^ Inc., San Diego, CA, United States) running the single read for 77 cycles. The generated sequences were then processed using proprietary DArT analytical pipelines. Approximately 2,500,000 sequences per barcode/sample were used in the marker call. Identical sequences were collapsed into fastqcall files followed by SNPs calling using the proprietary algorithm DArTsoft14. Finally, the SNP markers were mapped with the pea reference genome sequence assembly (Kreplak et al., 2019).

The leaf samples of the panel were outsourced to the LGC Genomics GmbH (Berlin, Germany) for GBS SNP discovery. The GBS genotyping was conducted following the procedure by Elshire et al. (2011) with some modifications. In brief, the DNA samples (200 ng) of the individual genotypes were digested with *Pst*I and *Msp*I restriction enzymes and ligated with unique 4–8 sequence barcode adapters. Equal aliquots of adapter-ligated DNA samples were pooled in a single tube to produce 59-plex libraries. The pooled DNA was amplified with the sequencing primer and purified using a QIAGEN PCR purification kit. The purified DNA was quantified and sequenced with the NextSeq 500/550 v2 (Illumina, San Diego, CA, United States) with 75 base pair (bp) single read. Demultiplexing of samples with barcode adapters ligated to individual DNA samples and verification of restriction site was processed using the in-house Illumina bcl2fastq v2 software package. The barcode sequences were removed from the read sequences, and the reads were trimmed using the in-house trimming software, the Trimmomatic-0.33. The SNP polymorphism discovery of filtered reads was made by mapping to the pea reference genome assembly using the sequence alignment tool, the BWA-MEM version 0.7. The filtering of variants and the minimum read depth was applied according to the GBS-specific ruleset [i.e., read count/locus >8, minor allele frequency (MAF) >0.05, and the total number of fully covered SNPs ≥ 66% of samples].

### Genetic Diversity, Population Structure, and Kinship Analysis

The genetic diversity estimation parameters, such as polymorphism information content (PIC), Nei’s gene diversity, heterozygosity, and MAF of SNP markers generated from the DArTseq and GBS genotyping platforms were calculated separately using the Power Marker v 3.25 (Liu and Muse, 2005). In addition, a phylogenetic tree was constructed according to Nei’s standard genetic distance (Nei, 1972) based on the unweighted pair group method with arithmetic mean (UPGMA; Sneath and Sokal, 1973) using the TASSEL software package v.5 (Bradbury et al., 2007) and visualized through the web-based program iTOL v 4.3.2 https://itol.embl.de/ (Letunic and Bork, 2019).

For population structure analysis, the Bayesian model-based clustering algorithm was applied using STRUCTURE ver. 2.3 (Pritchard et al., 2000). The most optimum sub-groups and the membership probability of genotypes to the corresponding sub-groups were computed and retrieved according to Evanno et al. (2005) *via* the web-based Structure Harvester ver. 0.6.94 http://taylor0.biology.ucla.edu/structureHarvester/ (Earl and Vonholdt, 2012). The analysis was done with 10,000 burn-in periods and 40,000 Markov Chain Monte Carlo (MCMC) replications after the burn-in, assuming an admixture model with uncorrelated allele frequencies running for ten (1–10) hypothetical subpopulations, each repeated for five iterations. Bar plots for the sub-groups were determined using the web-page program Clumpak beta version (Kopelman et al., 2015). The pair-wise SNP kinship similarity matrix was computed using the centered identity-by-state (IBS) method according to Endelman and Jannink (2012) in TASSEL v.5, and the heatmap was drawn using the package *Superheat* (Barter and Yu, 2018) in R environment (R Core Team, 2020). The SNP density and distribution across chromosomes were estimated in a 1-Mbp-window size using the R package *rMVP* (Yin et al., 2021).

### Linkage Disequilibrium and Genome-Wide Association Study Analysis

The pair-wise LD between SNP markers from DArTseq and GBS was calculated as *r*^2^ values in TASSEL. The chromosome-wise LD was calculated from complete SNP markers, while genome-wide LD was estimated from pairwise comparisons in 1,000 sliding window size. The specific critical *r*^2^ value beyond which LD is due to true physical linkage was determined by taking the 95th percentile of *r*^2^ data of the unlinked marker pairs (Breseghello and Sorrells, 2006). The maximum and half decay were estimated as physical distance and an LD decay curve was fitted with a smoothing spline regression line at the genome level, following the procedure by Hill and Weir (1988) using the R package *genetics* (Marroni et al., 2011). The GWAS analysis was performed using the multi-locus model, Fixed and random model Circulating Probability Unification (FarmCPU; Liu et al., 2016) available in the R package GAPIT v.3 (Wang and Zhang, 2021). The exploratory significant threshold of *P* ≤ 0.0001 [−log_10_ (*P*-value) ≥4] and Bonferroni threshold adjusted for multiple marker tests at *P* ≤ 0.05 were used to report significant marker-trait associations. The Bonferroni test was estimated with the formula: −log_10_ (α/*m*), where α is the overall false positive threshold (0.05) and *m* is the number of markers used for the GWAS analysis (6,902 SNPs). Thus, the Bonferroni threshold was set at −log_10_ (0.05/6,902) = 5.14, which corresponded to a *p*-value of 7.24e-6. The cryptic relatedness matrix (K) of genotypes based on the SNP markers was included in the GWAS analysis to control the familial kinship. Including equal numbers of principal components (PCs) across the studied traits created either inflation (false-positives, type-I error) or overcorrection (false-negatives, type-II error) of the GWAS results based on the produced quantile-quantile (Q-Q) plots. Because of this, Q-Q plots were first generated with different numbers of PCs and were used to select the appropriate number in the GWAS model based on the trait’s performance. A range of 1–3 PCs sufficiently captured the confounding effects of the population structure without overcorrections during the analysis.

### Phenotypic Data Analysis

After outlier testing and initial data cleaning for any possible recording errors, BLUP and other variance components were estimated from the historical unbalanced data sets recorded for different phenotypic traits using the package ASReml v. 4 (Butler et al., 2017), fitting a linear mixed model with the formula:


$${\text{Y}}_{\text{ijk}}=\,\mu +{\text{Gen}}_{\text{i}}+{\text{Rep}}_{\text{k}}\,\left({\text{Env}}_{\text{j}}\right)+{\text{Env}}_{\text{j}}+{\text{Gen}}_{\text{i}}\times {\text{Env}}_{\text{j}}+\varepsilon_{\text{ijk}}$$

where Y_ijk_ is the phenotypic value for **i**th genotype in ***k***th replicate at *j*th environment; μ is the overall mean effect; Gen_i_ and Env**_j_** are the effects from the *i*th genotype and *j*th environment, respectively; Rep_k_ (Env_i_) and Gen_i_ × Env_j_ are the effects of ***k***th replicate at the *j*th environment, and the genotype by environment interaction, respectively; and ε_ijk_ is the effect of the error associated with the *i*th genotype, *j*th environment, and ***k***th replicate. Both genotypes and environments were treated as random effects. The significance level of the genetic and G × E interaction variance components was computed from the log-likelihood ratio test based on the full and reduced models. Broad-sense heritability was estimated from the genotypic, G × E interaction and error variances using the following formula:


$$H^{2}=\frac{\sigma^{2}\,\text{g}}{\sigma^{2}\,\text{g}+\left(\sigma^{2}\,\text{ge}/n\,E\,n\,v\right)+\left(\sigma^{2}\,\text{e}/\left(n\,E\,n\,v\,X\,n\,R\,e\,p\right)\right)}$$

where σ^2^_g_ is the genotypic variance, σ^2^_e_ is the error variance, σ^2^_ge_ is the G × E interaction variance, and *n*Rep and *n*Env are the numbers of replicates and environments, respectively. Pearson’s correlation between BLUP estimated phenotypic values of traits was computed using *cor* function in R environment.

## Results

### Diversity Arrays Technology Sequencing and Genotyping-by-Sequencing Genotyping Performance

**FIGURE 1:**
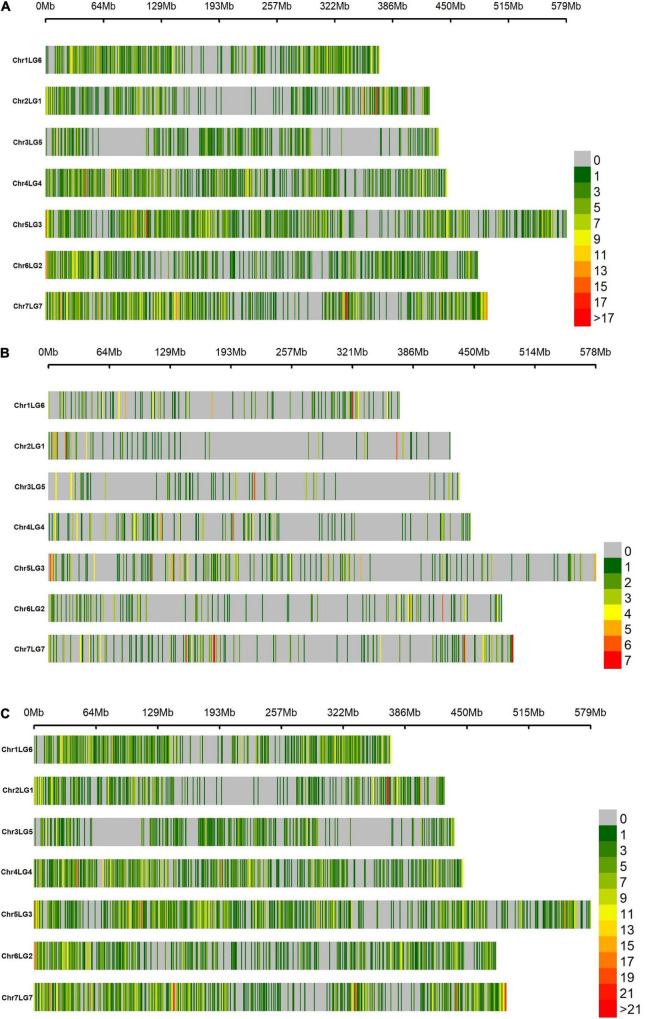
Density distribution of single nucleotide polymorphism (SNP) markers in a 1 Mbp window size across the seven chromosomes of wrinkled vining pea generated from **(A)** diversity arrays technology sequencing (DArTseq), **(B)** genotyping-by-sequencing (GBS), and **(C)** DArTseq and GBS combined. The assigned numbers of chromosomes and linkage groups are according to Neumann et al. (2002).

The DArTseq and GBS high-throughput genotyping methods were implemented to generate SNP markers from the current 188 wrinkled vining pea genotypes. A total of 9,306 SNP markers were produced from DArTseq, of which 61.9% (5,764 SNPs) reached the threshold applied for quality checking (i.e., MAF > 0.05 and missing values per genotype <0.1). From GBS, the overall SNP markers discovered across all samples and with a minimum read count of eight/locus were 13,528 and 5,784, respectively. However, only 1,138 (8.4%) markers reached the same quality threshold applied to the earlier sequencing method. Hence, the number of quality SNP markers generated from DArTseq was higher than GBS by more than five-fold. Notwithstanding, the SNPs discovered from the two genotyping approaches appeared to have a similar distribution pattern across chromosomes (Supplementary Figures 1A,B). For instance, the chromosome chr5LG3 contributed the highest markers with 1,062 and 232 SNPs from DArTseq and GBS, respectively. On the contrary, the smallest SNP markers were extracted from chr3LG5, with only 401 by DArTseq and 80 SNPs from GBS. The DArTseq SNP markers were uniformly distributed across the genomic regions covering all chromosome regions with minimal gaps (Figure 1A). However, the GBS genotyping had many abandoned chromosome regions due to the limited number of the generated SNP markers (Figure 1B). The density and coverage of SNPs are much better when combining the markers genotyped by the two used sequencing methods (Figure 1C).

### Genetic Diversity, Structure, and Clustering Analysis

The two sequencing approaches similarly estimated the existing genetic variation within the wrinkled vining pea genotypes. The small numbers of SNP markers generated from GBS equally quantified the existing allelic variation with the DArTseq markers (Supplementary Table 2).

**FIGURE 2:**
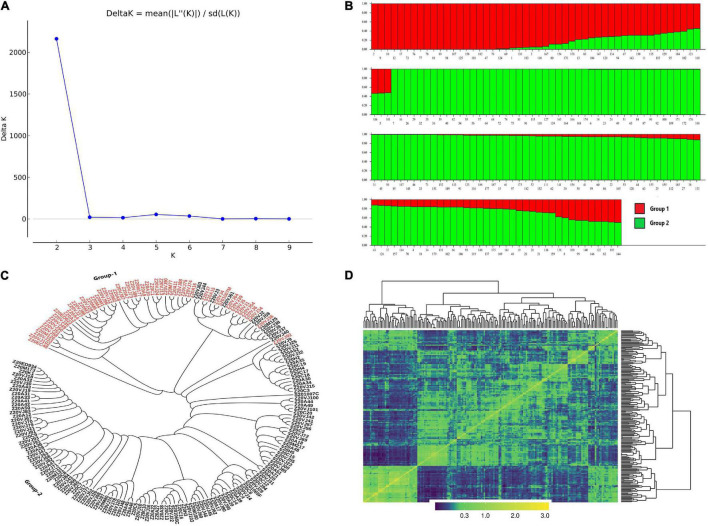
Genetic structure and clustering analysis of 188 wrinkled vining pea genotypes based on 9,602 SNP markers. **(A)** Inference of optimal sub-groups with the Bayesian clustering model in STRUCTURE. **(B)** Bar plot for the genetic composition of 188 wrinkled vining pea genotypes in the two STRUCTURE-inferred sub-groups. **(C)** Unweighted pair group method with the arithmetic mean (UPGMA)-based clustering of genotypes. **(D)** Heatmap of genotypes based on the identity-by-state (IBS) kinship similarity matrix.

The SNPs from the two sequencing methods were pooled together for the structure and clustering analysis. The Bayesian model clustering approach conducted in STRUCTURE identified two optimal sub-groups in the current panel (Figures 2A,B). Congruently, the unweighted pair group method with the arithmetic mean (UPGMA) clustering method grouped the majority of varieties separately into the two STRUCTURE-inferred sub-groups (Figure 2C). The heatmap for the pairwise SNP kinship similarity matrix based on the centered identity-by-state (IBS) method made two clear subgroups and aligned with other abovementioned genetic stratification analysis methods (Figure 2D). The first sub-group comprised 53 genotypes, while the rest 135 were encompassed in sub-group two (Figure 2B and Supplementary Table 1). The sub-group one comprised Findus and Birds Eye varieties and breeding lines together with lines that are identified as resistance sources for DM and root rot, whereas the sub-group two incorporated the reference line susceptible to DM together with other varieties and breeding lines from Findus. The majority (47) of wrinkled vining pea genotypes clustered in the first sub-group were Findus-originated breeding lines, and the two known resistance varieties to DM and root rot. The other four historical varieties developed from the Findus breeding program completed this sub-group. The three historical wrinkled vining pea varieties and six breeding lines from Birds Eye clustered together in the second sub-group with the other 126 breeding lines developed from the Findus breeding program.

### Phenotypic Variation and Heritability

**FIGURE 3:**
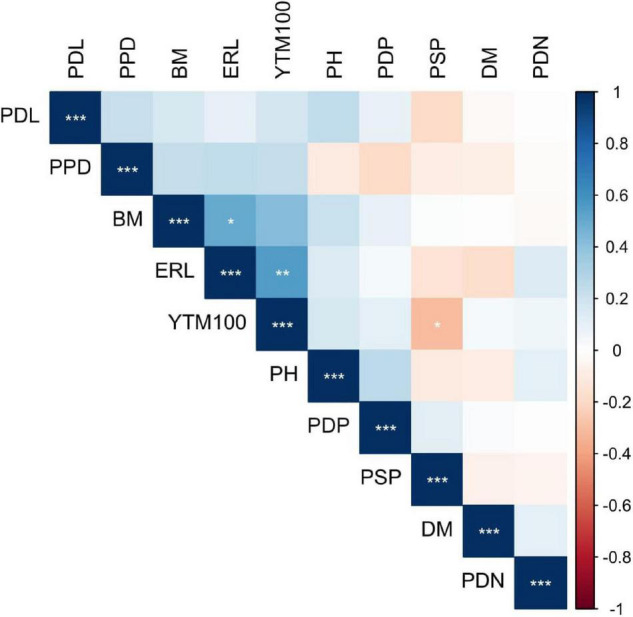
Person’s correlation coefficient and level of significance between best linear unbiased prediction (BLUP) estimated values of agronomic and downy mildew (DM) resistance traits of wrinkled vining pea genotypes. *P < 0.05, **P < 0.01, ***P < 0.001.

The analysis of variance for the two-decades-long recorded field data revealed a highly significant variation between genotypes and the genotype by environment (G × E) interaction for all agronomic and downy mildew resistance traits included in the current study (Table 1). The broad-sense heritability ranged from 0.23 recorded for adult plant resistance to DM to 0.99 for ERL. The broad-sense heritability for PH, YTM100, and PDP was 0.87, 0.65, and 0.81, respectively (Table 1). A normal frequency distribution was observed for all studied traits except ERL and DM resistance that exhibited bi-modal and skewed-left types of distribution, respectively (Supplementary Figure 2). Wrinkled vining pea yield had a significant positive correlation with earliness class, where the latter varieties and lines have a higher yield, but there is a significant negative correlation of wrinkled vining pea yield with the PSP (Figure 3).

**TABLE 1:** Estimated genetic variance, genotype-by-environment (G × E) interaction variance and residual variance components, and broad-sense heritability of agronomic and downy mildew resistance traits of wrinkled vining pea genotypes.

	PH	BM	ERL	YTM100	PDP	PDN	PDL	PPD	PSP	DM
Mean	58.08	68.34	9.55	5.33	3.63	1.83	55.34	6.28	0.23	96.41
σ^2^g	26.89	13.58	23.82	0.54	0.19	0.02	5.19	0.18	0.004	4.55
σ^2^ge	7.88	39.15	0.50	0.58	0.09	0.008	6.46	0.07	0.005	29.93
σ^2^e	14.36	26.71	0.003	0.45	0.27	0.02	13.14	0.27	0.001	26.71
*H* ^2^	0.87	0.40	0.99	0.65	0.81	0.86	0.61	0.83	0.60	0.23

*H^2^, broad-sense heritability; σ^2^g, genetic variance; σ^2^ge, genotype-by-environment interaction variance; σ^2^e, residual variance components. PH, plant height; BM, biomass; ERL, earliness; DM, downy mildew; PDL, pod length; PDP, number of pods per plant; PDN, Number of pods per node; PPD, number of peas per pod; YTM100, Wrinkled vining pea yield; PSP, percent of small wrinkled vining peas. Both the genetic and G × E interaction variance was significant at P < 0.0001 for all traits.*

### Linkage Disequilibrium

**FIGURE 4:**
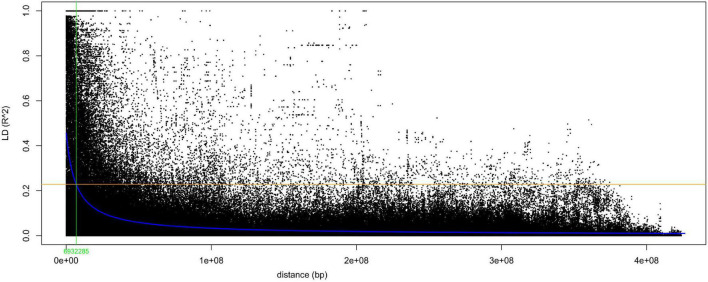
Genome-wide linkage disequilibrium (LD) decay curve in a scatter plot of pairwise SNPs *r*^2^ values against the physical distance (bP). The blue curve line is the smoothing spline regression model fitted to LD decay, the horizontal orange solid line is the half-LD decay of the genome (*r*^2^ = 0.23), and the vertical green solid line is the genetic distance (6.93 Mbp) of the intersect between the *r*^2^ value of the half-LD decay and the LD decay curve.

The individual chromosome linkage disequilibrium (LD) was calculated from the whole set of SNPs pairwise comparison. However, the genome-wide LD was estimated from 6,401,500 pairwise comparisons created from 6,902 SNP markers included in 1,000 sliding window sizes. The LD was varied across chromosomes with a mean *r*^2^ value ranging from 0.05 in chr4LG4 to 0.09 in chr6LG2, in which 29 and 25% of the pairwise LD comparisons were significant at *P* < 0.01, respectively (Supplementary Table 3). The mean *r*^2^ value of the genome was 0.034, with 33% of the pairwise LD comparison being significant. The specific critical *r*^2^ value beyond which LD is due to true physical linkage was estimated at 0.30, and the intersection with the LD decay curve was at 3.59 Mbp. The genome-wide maximum LD decay was started at r^2^ of 0.45 and reached the half decay at 0.22 (Figure 4). The genome-wide physical distance in which the LD reached half decay was estimated at 6.93 Mbp. The physical distance of half decay was different across chromosomes, in which the most rapid was chr1LG6 with 5.92 Mbp, while chr6LG2 was the slowest with 13.11 Mbp (Supplementary Figure 3).

### Genome-Wide Association Study Analysis

**FIGURE 5:**
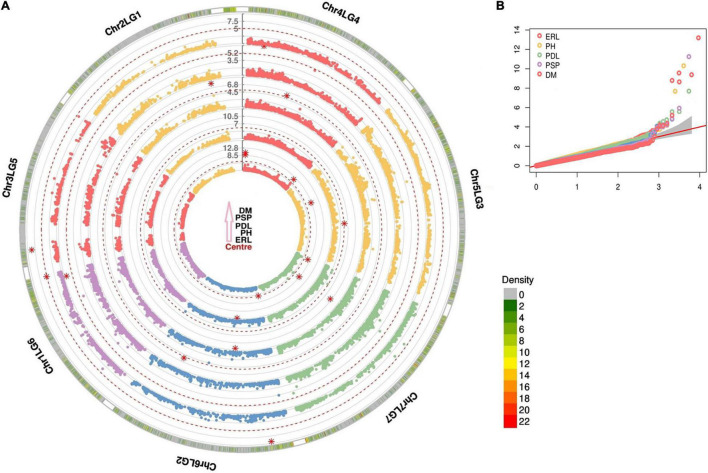
Circular Manhattan **(A)** and Q-Q plots **(B)** of six different traits from fixed and random model circulating probability unification (FarmCPU)-genome-wide association study (GWAS) model incorporating the first two PCs and cryptic relatedness as covariates. The red dash lines and red stars are the Bonferroni threshold and MTAs above the Bonferroni threshold, respectively. Earliness (ERL), plant height (PH), pod length (PDL), percent of small wrinkled vining peas (PSP), downy mildew (DM).

A total of 6,902 SNP markers encompassing 5,764 and 1,138 SNPs from DArTseq and GBS, respectively, were applied for the current GWAS analysis. Forty-eight significant marker-trait associations (*P* < 0.0001), of which 26 are with the Bonferroni threshold (*P* < 7.24e-6), were identified for all traits except for biomass and the number of pods per node (Supplementary Table 4 and Supplementary Figure 4). From the significant markers, the majority of SNP markers (42) were contributed from DArTseq, while the other six SNPs were from the GBS. The most MTAs were detected for ERL with nine followed by PH and PDL with eight and seven MTAs, respectively (Supplementary Table 4). Highly significant MTAs were identified for the trait ERL on chromosomes chr4LG4 (three), chr5LG3, chr6LG2 (two), and chr7LG7 (two) above the Bonferroni threshold, while MTAs for PH was discovered on chr7LG7, chr6LG2, chr5LG3, and a scaffold (SCAFFOLD04097) (Table 2). The SNP markers from chr2L1, chr4LG4, chr7LG7, and scaffolds (two) were identified with a significant association for wrinkled vining pea yield. Six MTAs were detected with a significant association for the PSP on chromosomes chr1LG6, chr2LG1, chr4LG4, chr7LG7 (two), and a scaffold. Highly significant MTAs were identified for adult plant resistance to DM on chromosomes chr1LG6, chr3LG5, and chr6LG2 above the Bonferroni corrected *p*-value (Figure 5). The Bonferroni threshold MTAs were detected for pod-related traits such as PDP on chr3LG5, PPD on chr7LG7 and chr5LG3, and PDL on chr2LG1, chr4LG4, and chr6LG2.

**TABLE 2:** List of above Bonferroni threshold significant SNP markers associated with agronomic and downy mildew resistance traits in wrinkled wining pea.

Trait	Chromosome	Marker	Source	Position	*P*-value	MAF	FDR	Effect
DM	Chr1LG6	3552605| F| 0-58:A > T-58:A > T	DArTseq	3.67E + 08	6.19E-06	0.461538	0.014247	−0.54437
	Chr3LG5	3559062| F| 0-22:G > T-22:G > T	DArTseq	41574379	2.36E-09	0.130177515	8.14E-06	1.794657786
	Chr6LG2	5943381| F| 0-59:G > T-59:G > T	DArTseq	54772562	4.49E-10	0.236686391	3.10E-06	−1.054403734
ERL	Chr4LG4	3548552| F| 0-33:T > A-33:T > A	DArTseq	5982791	1.08E-07	0.047337278	0.00018689	−6.262665302
	Chr4LG4	3549425| F| 0-57:T > C-57:T > C	DArTseq	6009062	5.78E-10	0.053254438	1.99E-06	1.337810786
	Chr4LG4	3553742| F| 0-43:G > C-43:G > C	DArTseq	4.38E + 08	1.03E-06	0.43787	0.001155	0.585533
	Chr5LG3	3563808| F| 0-61:C > T-61:C > T	DArTseq	177249420	1.50E-07	0.133136095	0.000206977	1.243221095
	Chr6LG2	3567609| F| 0-34:A > T-34:A > T	DArTseq	2581529	1.65E-06	0.269231	0.001425	0.659698
	Chr6LG2	5938969| F| 0-33:T > C-33:T > C	DArTseq	164655129	1.54E-14	0.233727811	1.06E-10	2.576023502
	Chr7LG7	3561951| F| 0-53:C > T-53:C > T	DArTseq	13684526	1.17E-06	0.378698	0.001155	0.603614
	Chr7LG7	3544639| F| 0-11:T > C-11:T > C	DArTseq	234618351	6.68E-08	0.133136095	0.000153742	−0.916961411
PDL	Chr2LG1	5938535| F| 0-18:G > T-18:G > T	DArTseq	389058072	3.06E-08	0.103550296	0.000211521	−1.014460371
	Chr4LG4	3546729| F| 0-57:C > A-57:C > A	DArTseq	2.34E + 08	1.56E-06	0.428994	0.00538	0.482608
	Chr6LG2	3547339| F| 0-61:G > A-61:G > A	DArTseq	3.71E + 08	6.77E-06	0.286982	0.015575	0.550724
PDP	Chr3LG5	S3LG5_7947727	GBS	7947727	1.83E-11	0.073964497	1.26E-07	−0.386833331
PH	Chr5LG3	S5LG3_238098849	GBS	2.38E + 08	1.96E-06	0.071006	0.003389	−2.54068
	Chr6LG2	S6LG2_472113027	GBS	472113027	2.36E-08	0.130177515	5.43E-05	2.070155861
	Chr7LG7	3565896| F| 0-60:G > C-60:G > C	DArTseq	148423594	1.38E-11	0.153846154	4.76E-08	−2.964130869
	SCAFFOLD04097	3540562| F| 0-39:T > C-39:T > C	DArTseq	DArTseq	4.10E-12	0.24556213	2.83E-08	4.424804552
PPD	Chr5LG3	4661147| F| 0-28:A > G-28:A > G	DArTseq	2.31E + 08	5.51E-06	0.186391	0.013047	0.080142
	Chr7LG7	41127127| F| 0-5:G > T-5:G > T	DArTseq	484994446	2.94E-08	0.142011834	0.000202986	−0.144075282
PSP	Chr1LG6	3546762| F| 0-59:A > G-59:A > G	DArTseq	3.55E + 08	1.30E-06	0.372781	0.00299	−0.01515
	Chr4LG4	3542939| F| 0-25:A > G-25:A > G	DArTseq	53259113	2.97E-12	0.121301775	2.05E-08	−0.044007921
	SCAFFOLD00066	3555121| F| 0-8:C > G-8:C > G	DArTseq	78412	1.99E-11	0.097633	6.85E-08	0.036651
YTM100	Chr2LG1	S2LG1_7763798	GBS	7763798	2.76E-06	0.201183	0.011664	−0.20777
	Chr7LG7	5958966| F| 0-20:G > T-20:G > T	DArTseq	4.4E + 08	3.38E-06	0.159763	0.011664	0.265625

*PH, plant height; ERL, earliness; DM, downy mildew; PDL, pod length; PDP, number of pods per plant; PPD, number of peas per pod; YTM100, wrinkled vining pea yield; PSP, percent of small wrinkled vining peas; MAF, Minor allele frequency; FDR, false discovery rate adjusted (FDR) p-value.*

**FIGURE 6:**
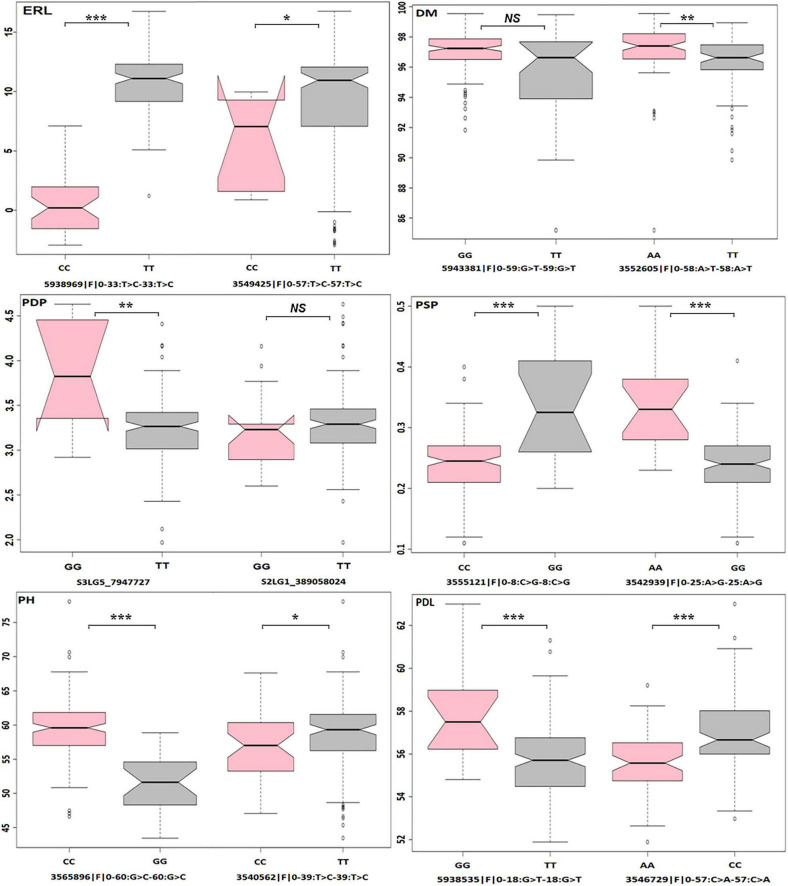
Allelic effects of the top two SNPs with significant marker-trait associations (MTAs) on the estimated BLUPs of phenotypic records of six traits. Earliness (ERL), downy mildew (DM), the number of pods per plant (PDP), percent of small, wrinkled vining peas (PSP), plant height (PH), pod length (PDL). *P < 0.05; **P < 0.01; ***P < 0.001; NS, non-significant.

The average values of individuals with the favorable allele of the topmost significant SNP marker *5938969| F| 0-33:T* > *C-33:T* > *C* (MAF = 0.23) on chr6LG2 significantly increased the days to maturity by 10.25, while the second topmost significant marker *3549425| F| 0-57:T* > *C-57:T* > *C* (MAF = 0.053) on chr4LG4 increased the plant’s maturity by 2.8 days (Figure 6). The favorable allele of *S3LG5_7947727* (MAF = 0.07) marker on chr3LG5 significantly increased the average numbers of pods per plant by one compared to the alternative allele. The favorable allele of marker *5938535| F| 0-18:G* > *T-18:G* > *T* (MAF = 0.10) on chr2LG1 increased the pod length by 2.18 mm.

## Discussion

### Single Nucleotide Polymorphism Markers Distribution and Genetic Diversity

Genetic diversity of crops is an essential part of breeding for the successful selection and development of new varieties that are advanced with certain characteristics of agronomic importance. A gene pool with rich genetic variation commonly leads to novel allele combinations with an enormous potential to enhance crop yield and to endure the recurrent biotic and abiotic stress due to climate change, thereby safeguarding food security. Nonetheless, a gene pool with narrow genetic diversity is susceptible to emerging pathogens and other restraints that lead to loss of productivity and declining adaptation to particular agroecology (Dyer et al., 2014; Gali et al., 2019).

The current panel comprises 188 wrinkled vining pea genotypes including varieties, breeding lines, and individuals with different resistance levels to DM and root rot. The panel was subjected to SNP genotyping with two different approaches (DArTseq and GBS). The result indicated that DArTseq was more efficient than GBS for high-throughput SNP discovery in the current wrinkled vining pea panel. The DArTseq sequencing method was discovered more than five-fold higher quality SNP markers than GBS. This is due to the higher sequence depth and usage of strict filtering criterion, which the earlier method applied that ultimately lead to generating higher numbers of quality SNPs with less missing data compared to the latter approach (Allan et al., 2020). The SNP markers from two sequencing platforms, however, enumerated the existing genetic diversity similarly. The SNP markers quantified the current genotypes with an intermediate level of genetic variation compared to the previous studies in pea (Burstin et al., 2015; Rana et al., 2017; Hanci, 2019). However, the higher genetic diversity reported by the abovementioned authors included pea accessions encompassing wild genotypes, landraces, and old cultivars collected worldwide. Jain et al. (2014) described the genetic diversity of 96 cultivars widely grown or used in breeding programs in the United States and Canada and reported a genetic variation in a similar range with the current study. The ten different phenotypic data scores for traits related to yield and DM resistance indicated a highly significant genetic variation among the studied wrinkled vining pea genotypes.

### Marker-Trait Associations

Considering the increments in the affordability of genotyping costs coupled with the availability of reference genomes, resequencing crop plants and exploring QTLs of quantitatively inherited traits are imperative for marker-assisted selection. However, the limited population size and the nature of unbalanced data sets have hindered the application of GWAS analysis in the widely applicable breeding-program-derived phenotypic data (Wang et al., 2012). Nonetheless, the mixed model’s statistical approaches have successfully utilized such historically recorded unbalanced phenotypic data to dissect the QTLs of various economically relevant traits in different crops. For instance, Wang et al. (2012) applied the balanced and unbalanced data sets recorded in breeding programs for GWA analysis of PH in spring barley breeding lines. They pointed out that the phenotypic scores from the unbalanced data sets led to a higher number of false-positive QTLs than the balanced one and suggested careful consideration of the population size and experimental design. However, Johnson et al. (2019) disputed this and specified that higher numbers of QTLs were identified with the balanced data than the unbalanced due to the false-negative QTLs masked on the later data type. Our study demonstrated that MTAs/QTLs could be successfully identified from breeding programs derived historical unbalanced data sets with the careful GWAS model selection, particularly when considering population structure effects.

The current study identified 48 SNP markers that are significantly associated with eight studied wrinkled vining pea traits related to agronomy and DM resistance. Some of the detected MTAs overlapped with previous studies conducted in pea and others could be the newly spotted QTLs. In fact, this study could be taken as a pioneer to use a complete panel of wrinkled vining pea genotypes aimed at the detection of QTLs for key traits with invaluable importance for the productivity of the crop. The DM, caused by the fungal pathogen *Peronospora viciae* f. sp. *pisi*, is one of the most important foliar diseases leading to a significant reduction in yield and quality of pea (Stegmark, 1994; Chang et al., 2013). The current study identified MTAs for adult plant resistance to downy on chromosomes chr1LG6, chr3LG5, chr6LG2, and chr7LG7. Minimal numbers of studies have been done to identify QTLs to adult plant resistance to DM resistance in pea before the availability of the pea reference genome, and these studies were mainly focused on the identification and validation of qualitatively inherited major genes for seedling resistance, *Rpv*, found on LG1 (Hanemann et al., 2017). This gene could not be found or validated on the current GWAS study due to different possible reasons, such as low allelic frequency, G × E interaction, epistatic interaction, minor/under-significant threshold QTL effect, gaps in the SNP coverage (Huang and Han, 2014), or non-availability of the gene on the current wrinkled vining pea panel. It was not easy to compare the currently detected QTLs with the results of these studies reported on other chromosome regions due to the different markers applied and the unavailability of physical positions of the markers on the reference genome. Our study could be taken as the first attempt to detect MTAs of adult plant resistance to downy mildew in wrinkled vining pea during the post-pea reference genome era.

This study identified nine MTAs for ERL, one of the most essential agronomic traits in wrinkled vining pea production, in which three were on chr4LG4, two each on chr6LG2 and chr7LG7, and one each on chr1LG6 and chr5LG3. The SNP marker *5938969| F| 0-33:T* > *C-33:T* > *C* on chr6LG2, particularly, had a highly significant effect on the ERL of wrinkled vining pea genotypes. Likewise, Desgroux et al. (2016) reported highly significant QTLs on chr6LG2 for the same trait from 175 dry pea genotypes. Furthermore, they reported ERL-related QTLs on chr5LG3 and chr7LG7. However, it was difficult to compare the exact positions of QTLs with our MTAs due to the different markers utilized and the absence of the physical position of these QTLs on the reference genome. Gali et al. (2019) also reported the MTAs on chr1LG6, chr4LG4, and chr6LG6 for days to flowering and on chr5LG3 for days to maturity.

Eight MTAs, of which three each on chr6LG6 and chr7LG7, one on chr5LG3, and one a scaffold, were detected for PH. The current study identified a Bonferroni significant MTA on chr5LG3 with the SNP marker *S5LG3_238098849* generated from GBS. Similarly, Gali et al. (2019) reported four MTAs on the same chromosome region for PH with the GBS-derived SNP markers produced from 135 field pea genotypes. In addition, Tar’an et al. (2003) on chr5LG3, and Desgroux et al. (2016) on both chr5LG3 and chr7LG7, reported QTLs for PH in field pea.

Numbers of pods per plant (PDP), pod length (PDL), and wrinkled vining number of peas per pod (PPD), were the three pod-related yield component traits included in the current GWAS study. Three significant MTAs were spotted for PDP, of which two were on chr2LG1 and the other one on chr3LG5. The GBS SNP marker *S3LG5_7947727* detected on chr3LG5 had particularly a significant effect on the pod number. Similarly, Tafesse et al. (2020) reported a multi-environment stable MTA for PDP on chr3LG5 from 135 diverse pea accessions, which are collected worldwide using an SNP marker generated from GBS. However, it is noteworthy to mention that the physical position of this marker (*Chr3LG5_216337201*, 216.3 Mbp) is far from the currently discovered marker (7.95 Mbp). Nonetheless, the authors reported other MTA on chr2LG1 (402.0 Mbp) with close proximity to the marker *S2LG1_389058024* (389.1 Mbp) identified in our study. The four MTAs detected for PPD were on chromosomes chr1LG6 (two), chr5LG3, and chr7LG7. Similarly, Huang et al. (2017) reported two QTLs on chr5LG3 for seed number per pod from the bi-parental linkage mapping of 107 field pea RILs. Nevertheless, it was difficult to compare the exact position of these two QTLs with the currently detected MTA on chr5LG3 due to the lack of a physical map and implementation of different markers. Since, to our knowledge, no previous attempts have been made for the trait PDL, the seven MTAs detected on chromosomes chr2LG1, chr3LG5, chr4LG4 (two), chr5LG3, chr6LG2, and chr7LG7 could be taken as the first report on genomic regions associated with the pod length of a vining pea.

The maturity of vining pea and tenderness is usually determined by a tenderometer that measures the shearing force needed to press immature pea seed (green peas) samples through a standard grid (Anderson and White, 1974). The tenderometer readings rise along seed maturation process, tenderness of immature pea seeds (green peas), when they are most suitable for freezing, is at tenderometer reading of 85–105 (Martin, 1981), and 100-tenderometer reading has been widely used for standard quality with acceptable texture quality of green peas for freezing. The genetic background of varieties influences this parameter along with the environment, maturity at harvesting, and post-harvest processing conditions (Edelenbos et al., 2001). Estimation of yield at a particular tenderometer reading is, therefore, essential to increase the yield of wrinkled vining pea genotypes with the required quality ranks. The proportion of small wrinkled vining peas is also a trait of interest since is used for higher rank products with increased tenderness (Salunkhe and Adsule, 1998). For the first time, this study identified SNP markers that are significantly associated with YTM100 on chr2LG1, chr7L7, chr4LG4, scaffolds (two), and for PSP on chr4LG4, chr1LG6, chr2LG1, chr7LG7 (two), and a scaffold. To our knowledge, no efforts have been made previously to detect the QTLs related to these valuable traits in vining pea production.

This study applied the GWAS approach to uncover candidate genetic variants or MTAs connected to the traits of economic importance for wrinkled vining pea production. Validations of the identified MTAs and further functional studies are prospects for the successful employment of marker-assisted selection. Furthermore, the identified SNP markers linked to the traits of interest would assist in the improvement of genomic selection models’ accuracy in the estimation of genomic estimated breeding values (GEBVs) of candidate progenies or breeding lines.

## Conclusion

The current study exploited 188 wrinkled vining pea genotypes to estimate the existing genetic variation and to detect the markers associated with various economically relevant traits in wrinkled vining pea production. Genotypes were sequenced for SNPs with both DArTseq and GBS approaches. The pattern of SNP distribution from these two sequencing platforms was uniform across chromosomes, though the number of SNPs generated from DArTseq was more than five times higher than the GBS. The two sequencing platforms similarly estimated the existing genetic variation. However, the higher quality-checked SNP markers generated from DArTseq were more powerful in the LD estimation and GWAS analysis. Hence, a higher number of SNP markers from DArTseq was identified with significant association to the studied traits. The current study detected several valuable MTAs *via* GWAS for eight traits related to agronomic and DM resistance using the wrinkled vining pea breeding-program-derived unbalanced phenotypic data sets recorded for the past two decades. We reported several novel MTAs for different economically relevant traits in wrinkled vining pea production for the first time, and these candidate markers could be validated and easily integrated for marker-assisted selection in the active breeding programs.

## Data Availability Statement

The genotypic data used for the findings of this study are available from authors upon reasonable request.

## Author Contributions

AC and AB conceived and designed the study. AA carried out the data analysis, curation, and first draft manuscript write-up. All authors contributed to the data interpretation, manuscript revision, and approval of the final version.

## Conflict of Interest

AB was employed by Nomad Foods Ltd. The remaining authors declare that the research was conducted in the absence of any commercial or financial relationships that could be construed as a potential conflict of interest.

## Publisher’s Note

All claims expressed in this article are solely those of the authors and do not necessarily represent those of their affiliated organizations, or those of the publisher, the editors and the reviewers. Any product that may be evaluated in this article, or claim that may be made by its manufacturer, is not guaranteed or endorsed by the publisher.

## Footnotes

1
http://www.fao.org/faostat/

2
https://www.diversityarrays.com/
