# Supplementary material for: Unraveling the Genetic Basis of Key Agronomic Traits of Wrinkled Vining Pea (Pisum sativum L.) for Sustainable Production
Source: Front Plant Sci. 2022 Mar 14;13:844450. doi: 10.3389/fpls.2022.844450 (PMC8964273; doi:10.3389/fpls.2022.844450)
Supplement: Supplementary file 2 [file Table_2.DOCX]

**Supplementary Table 2.** Genetic diversity analysis with SNP markers generated from DArTseq and GBS in 188 green pea genotypes.

| **Parameters** | **DArTseq** | | | **GBS** | | |
| --- | --- | --- | --- | --- | --- | --- |
|  | **Min** | **Max** | **Mean** | **Min** | **Max** | **Mean** |
| **Gene diversity** | 0.006 | 0.5 | 0.33 | 0.08 | 0.5 | 0.34 |
| **Polymorphism information content (PIC)** | 0.006 | 0.38 | 0.27 | 0.07 | 0.38 | 0.27 |
| **Minor allele frequency (MAF)** | 0.03 | 0.5 | 0.24 | 0.04 | 0.49 | 0.25 |
| **Heterozygosity** | 0 | 0.7 | 0.06 | 0 | 0.97 | 0.06 |
