# Supplementary material for: Unraveling the Genetic Basis of Key Agronomic Traits of Wrinkled Vining Pea (Pisum sativum L.) for Sustainable Production
Source: Front Plant Sci. 2022 Mar 14;13:844450. doi: 10.3389/fpls.2022.844450 (PMC8964273; doi:10.3389/fpls.2022.844450)
Supplement: Supplementary file 3 [file Table_3.DOCX]

**Supplementary Table** **3.** Mean and significance of pairwise LD and LD decay estimations across chromosomes and the genome using the two sequencing platforms.

| **Chromosomes** | **Mean LD (R^2^)** | **Significant LDs (p<0.001)** | **LD half-decay (bp)** |
| --- | --- | --- | --- |
| **Chr1LG6** | 0.06 | 0.29 | 5,916,721 |
| **Chr2LG1** | 0.08 | 0.31 | 9,067,257 |
| **Chr3LG5** | 0.08 | 0.25 | 8,121,914 |
| **Chr4LG4** | 0.05 | 0.29 | 4,579,970 |
| **Chr5LG3** | 0.06 | 0.24 | 8,762,830 |
| **Chr6LG2** | 0.09 | 0.25 | 13,108,987 |
| **Chr7LG7** | 0.06 | 0.30 | 7,529,618 |
| **Genome** (only DArTseq markers) | 0.029 | 0.44 | 7,529,618 |
| **Genome** (only GBS markers) | 0.033 | 0.33 | 8,098,698 |
| **Genome-wide** | 0.034 | 0.33 | 6,932,285 |
