# Supplementary material for: Unraveling the Genetic Basis of Key Agronomic Traits of Wrinkled Vining Pea (Pisum sativum L.) for Sustainable Production
Source: Front Plant Sci. 2022 Mar 14;13:844450. doi: 10.3389/fpls.2022.844450 (PMC8964273; doi:10.3389/fpls.2022.844450)
Supplement: Supplementary file 5 [file Data_Sheet_1.docx]

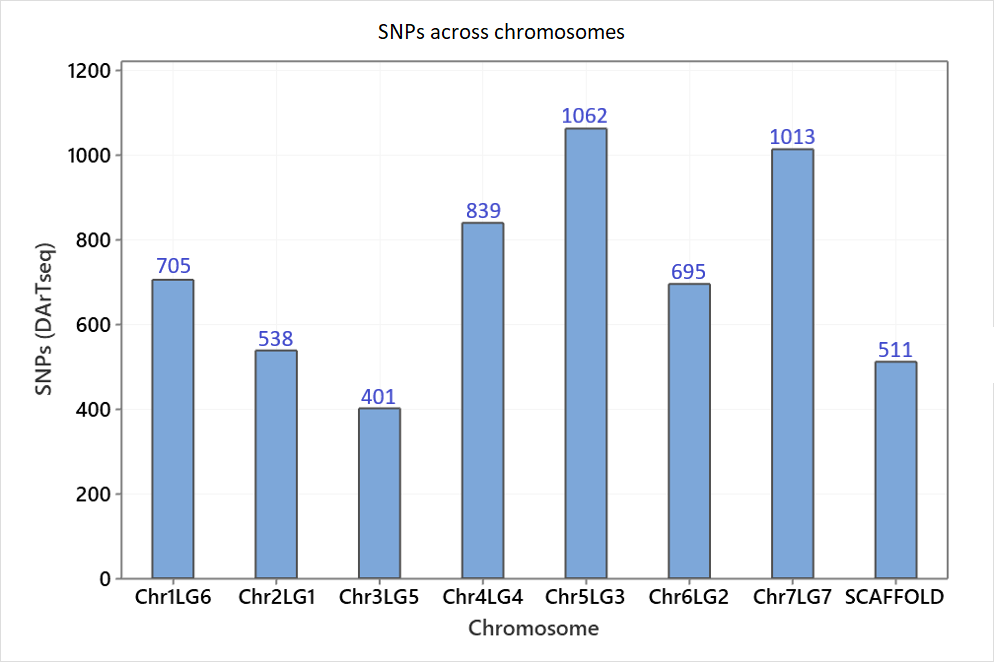

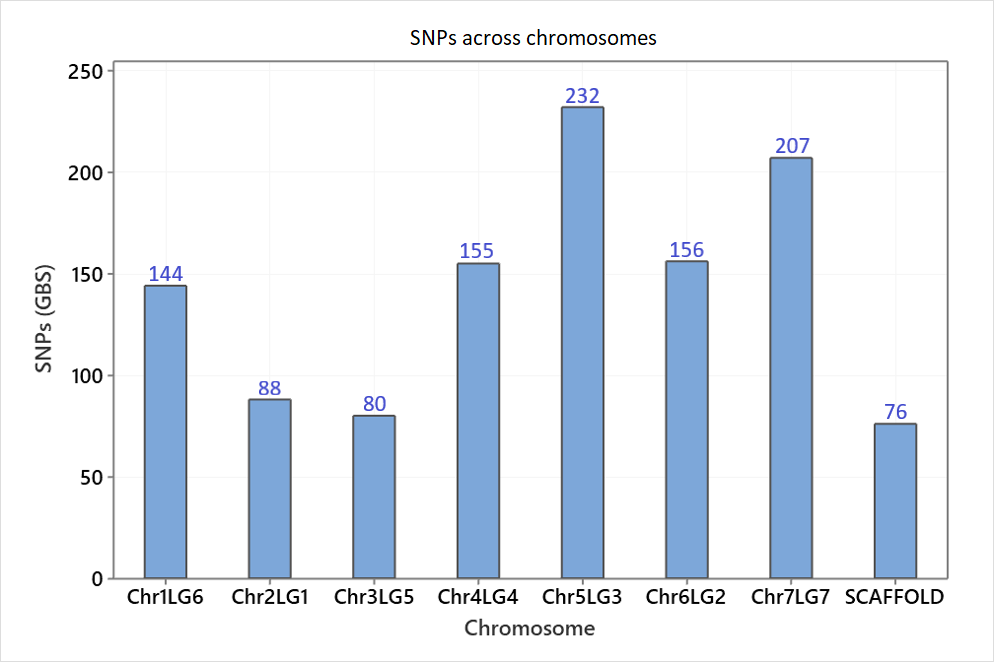


**B**

**A**

**Suppplementary Figure 1.** Distribution of SNP markers from DArTseq (**A**) and GBS (**B**) across the seven chromosomes and scaffolds generated from 188 green pea genotypes.


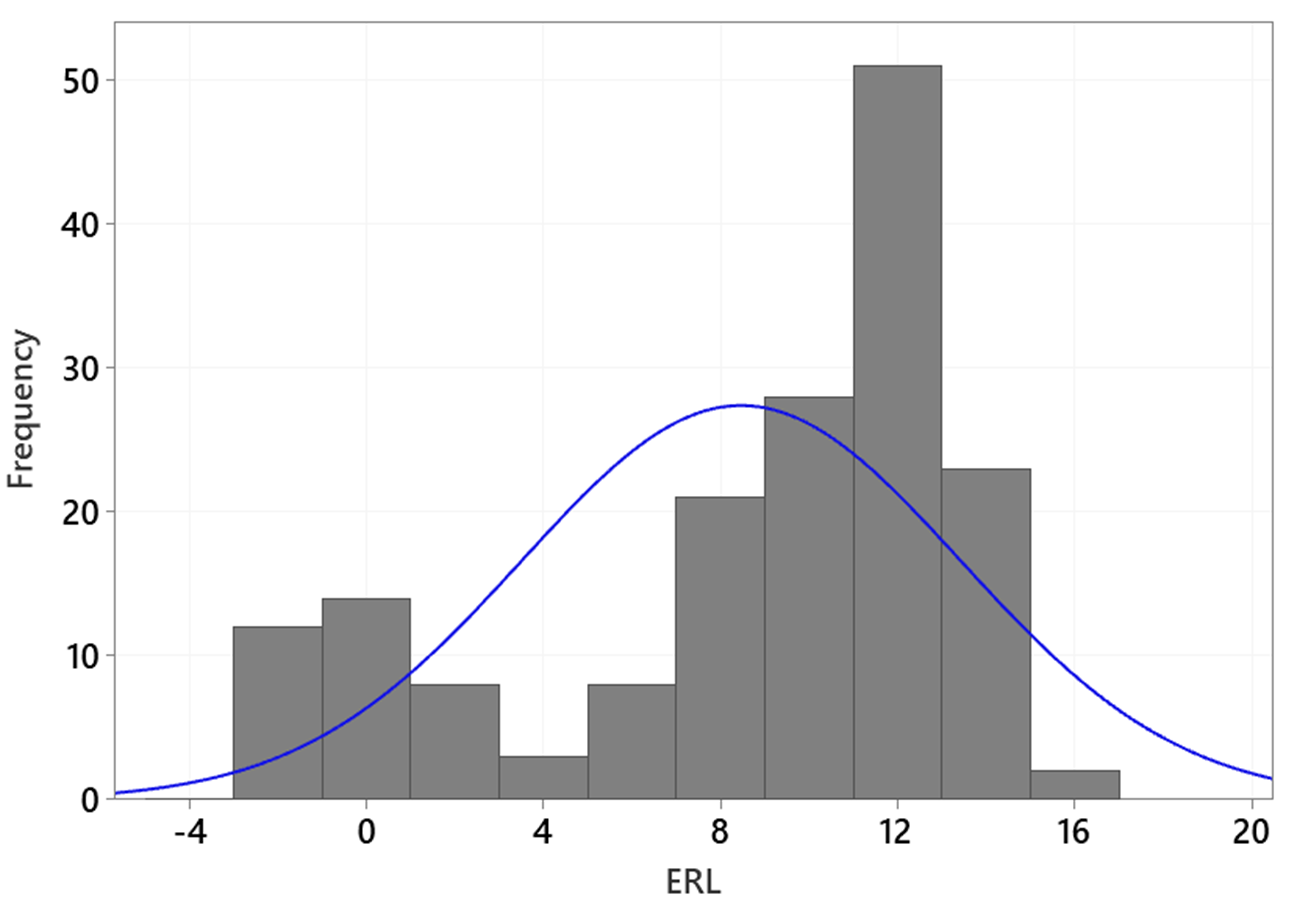

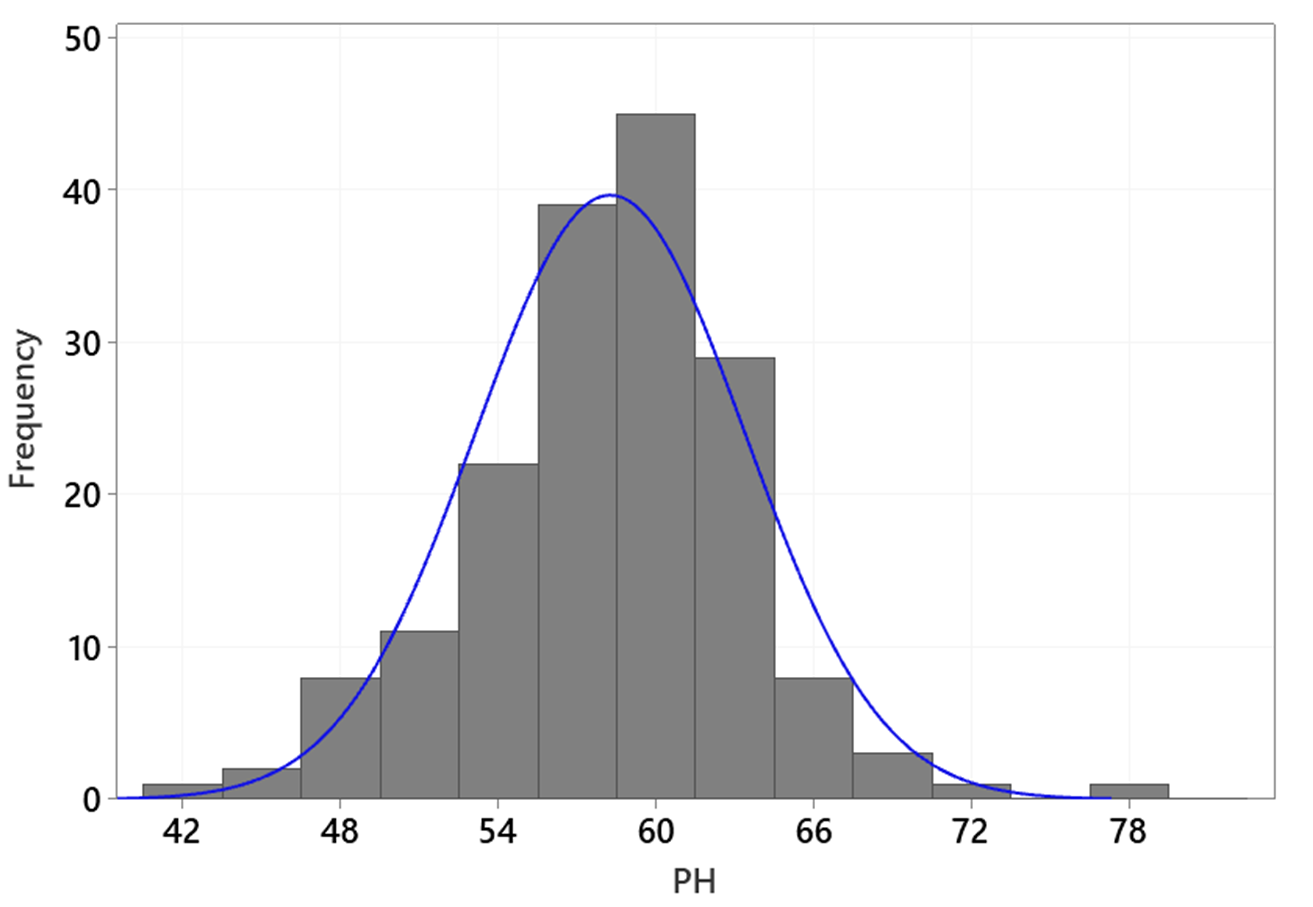


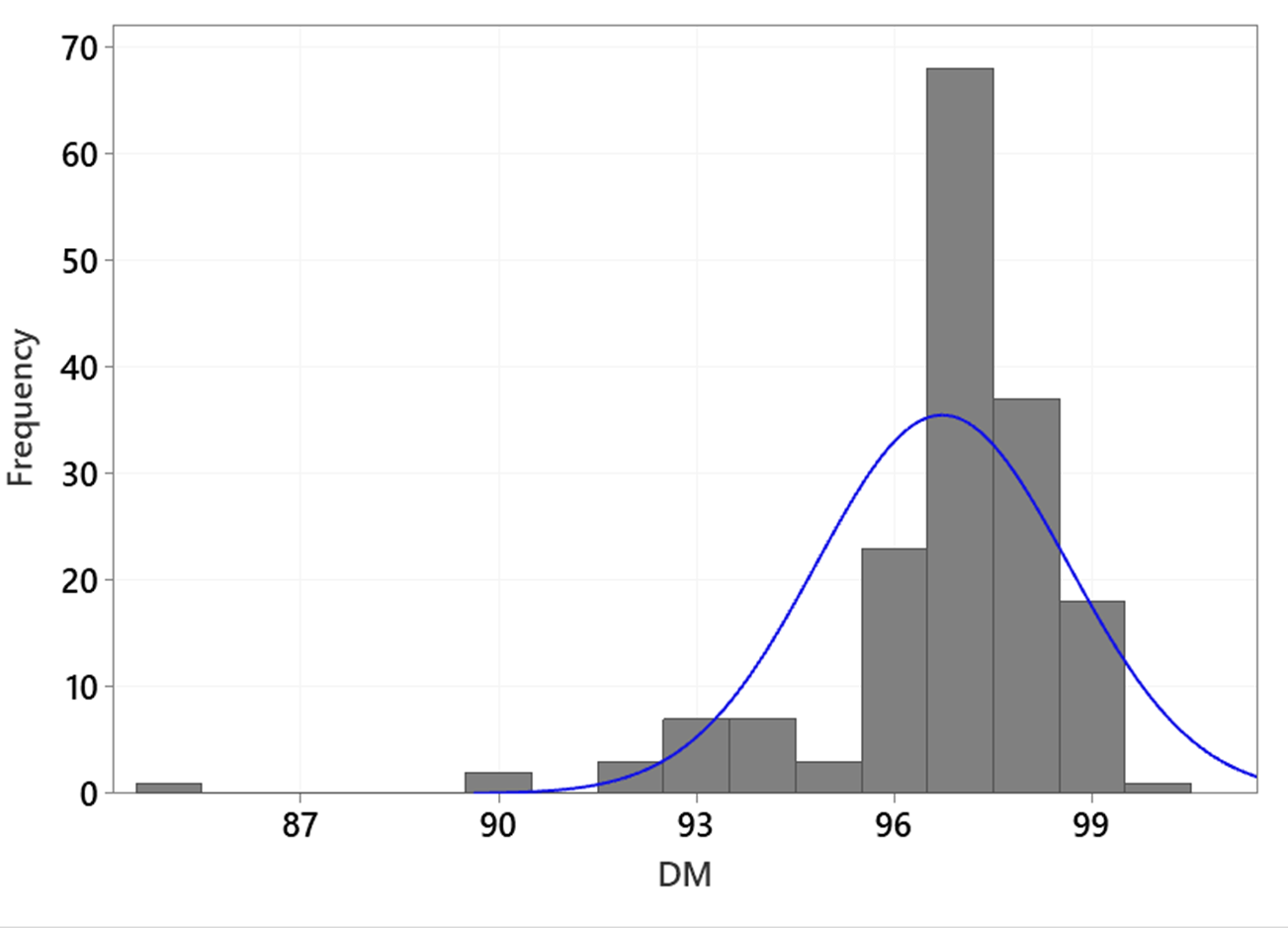

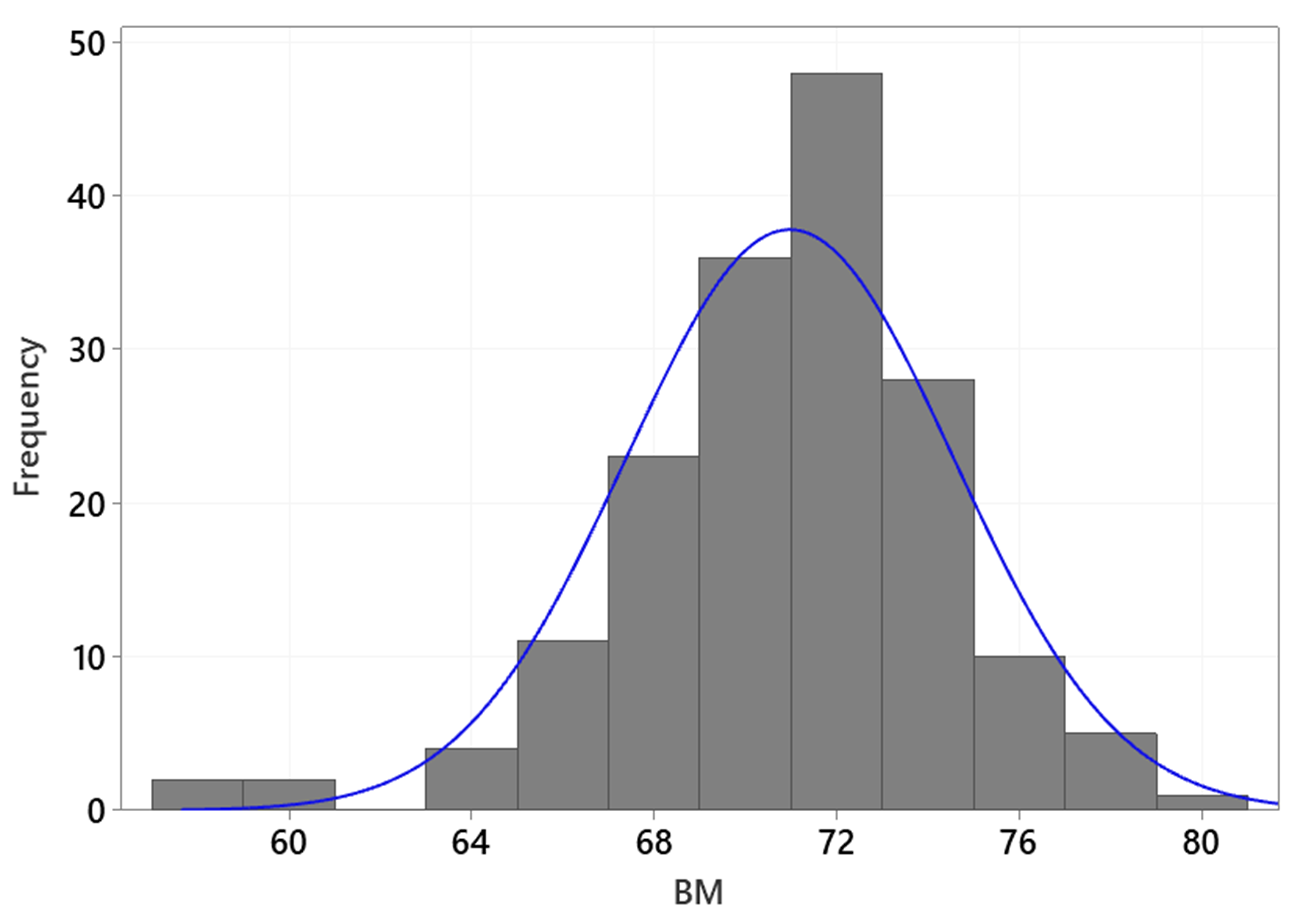


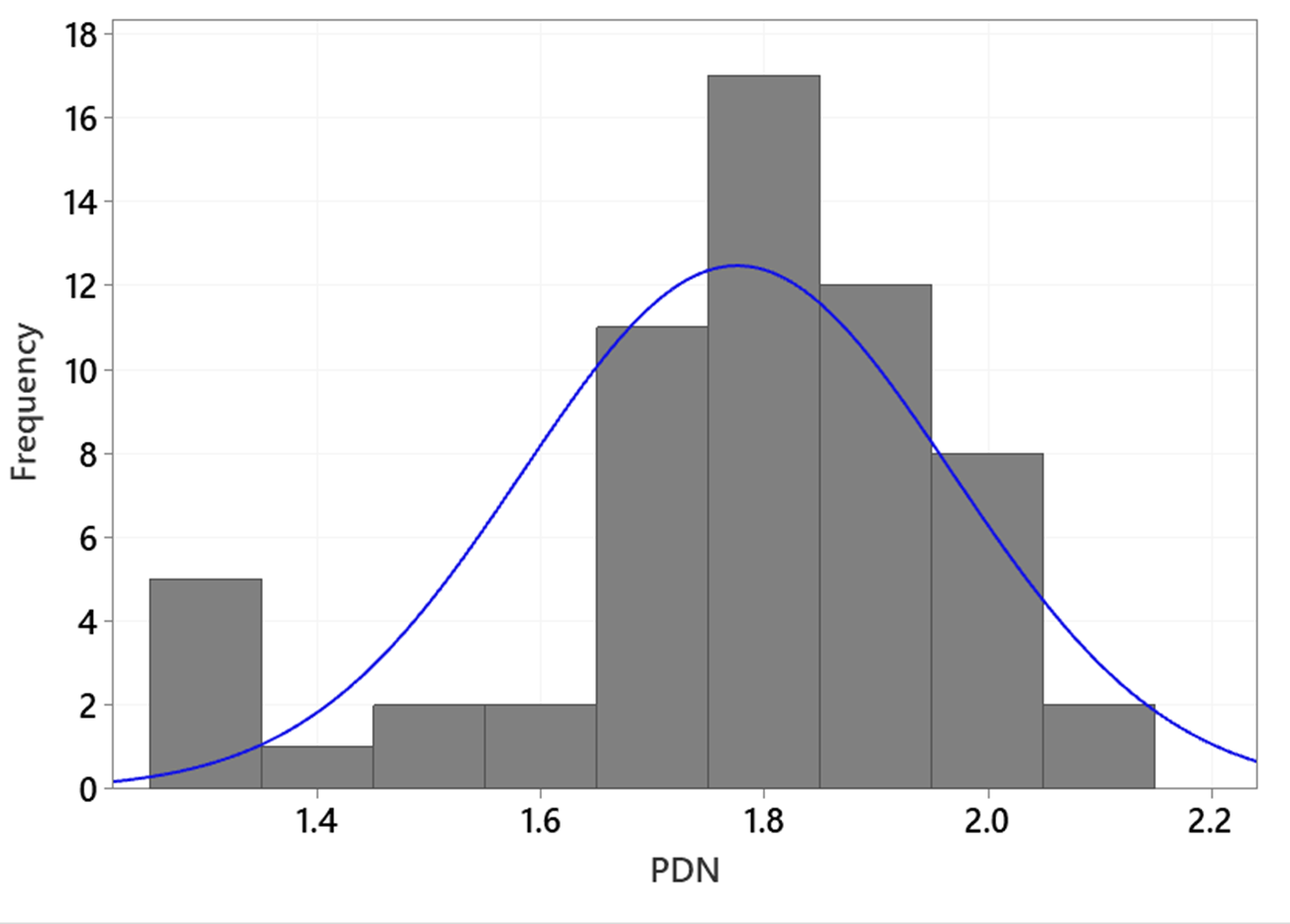

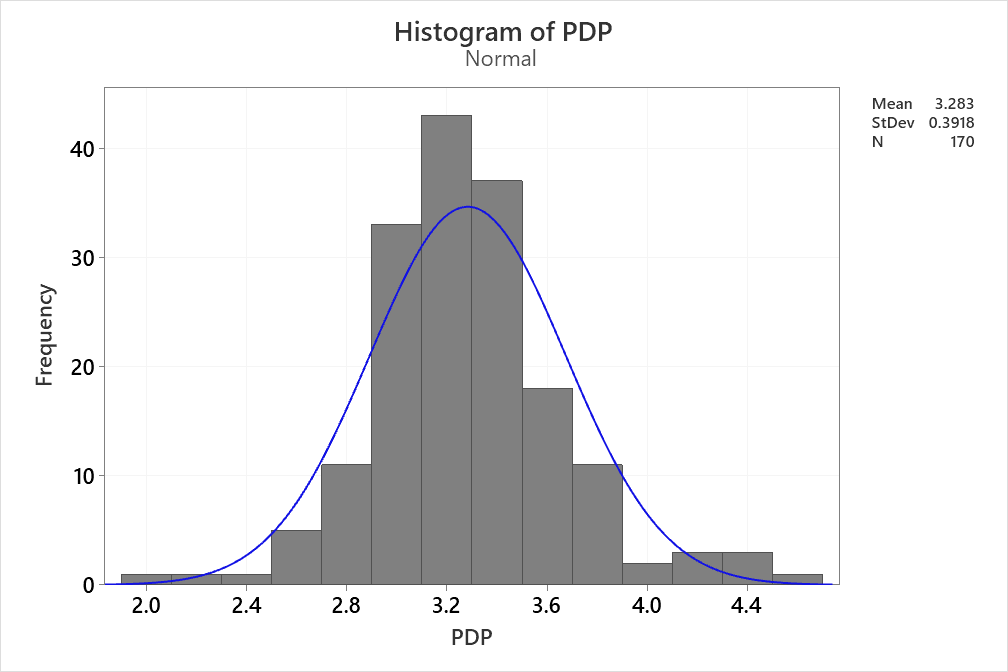


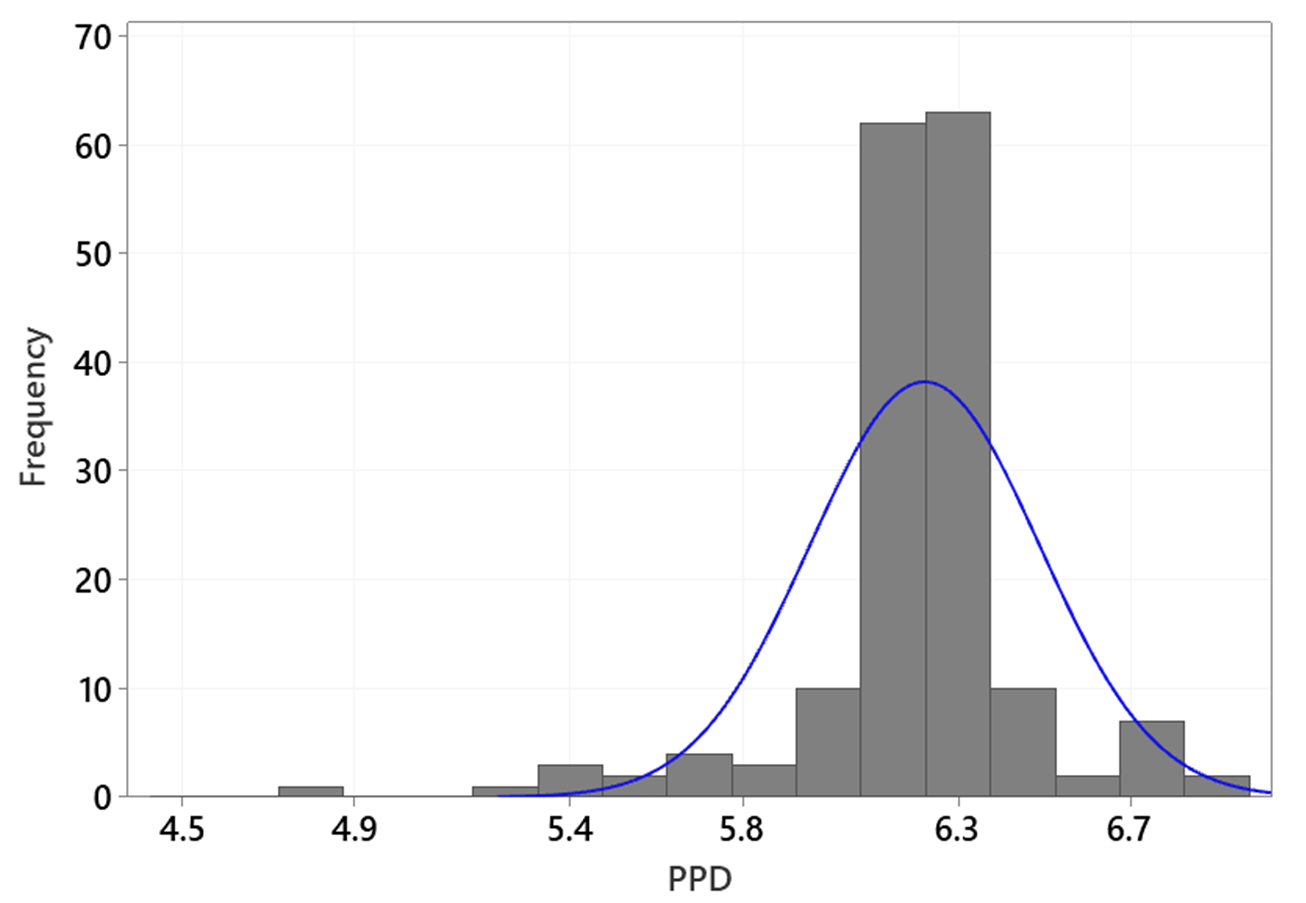

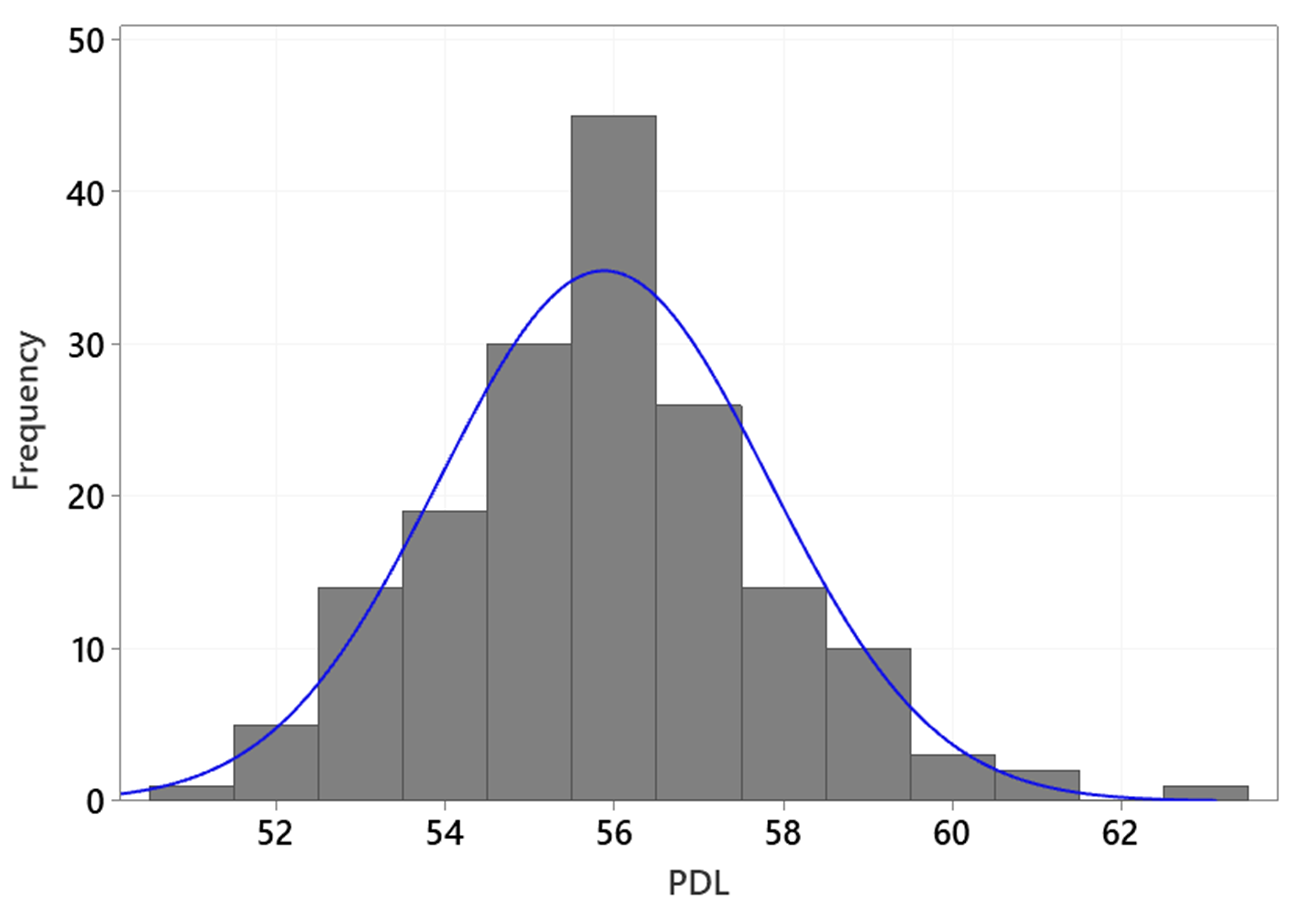


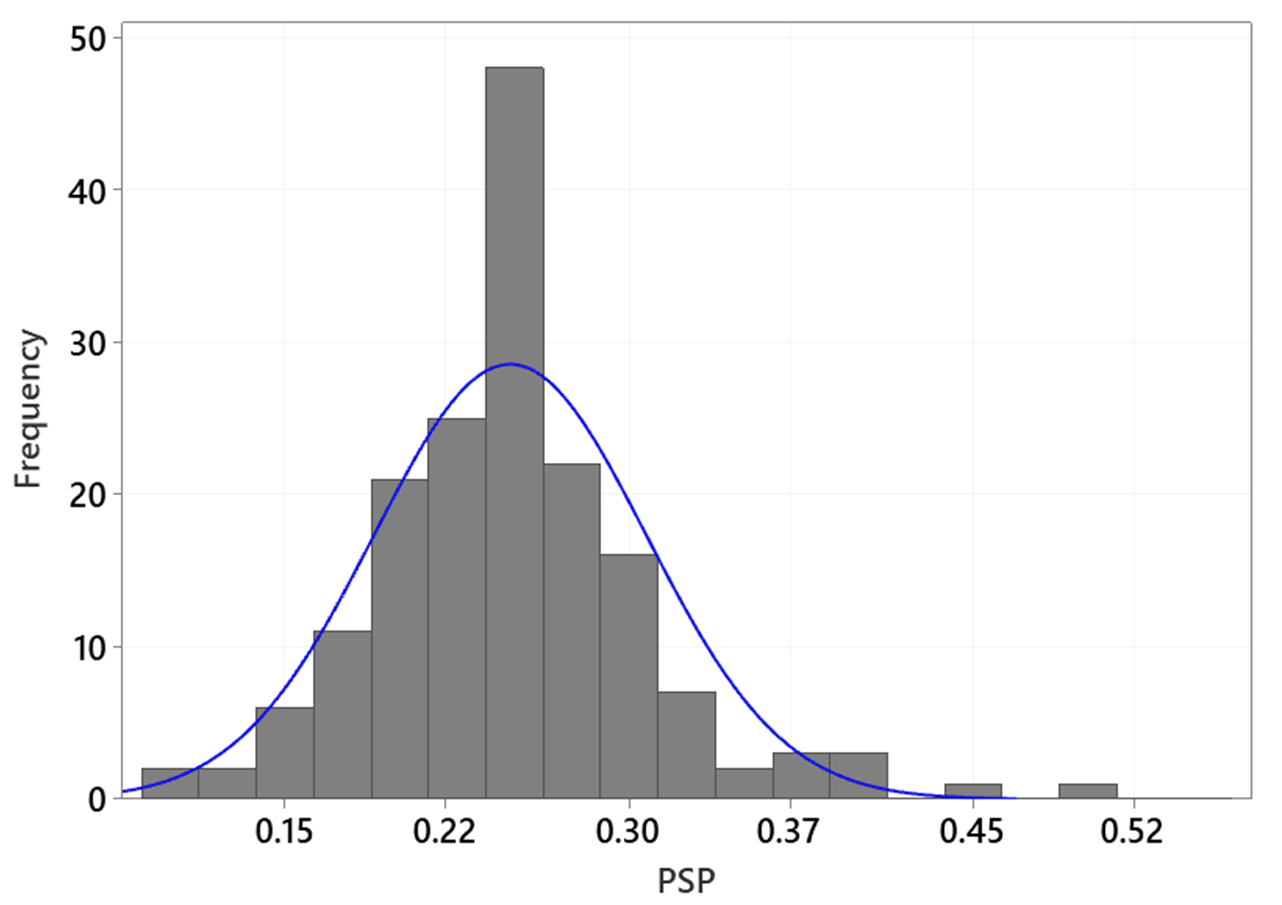

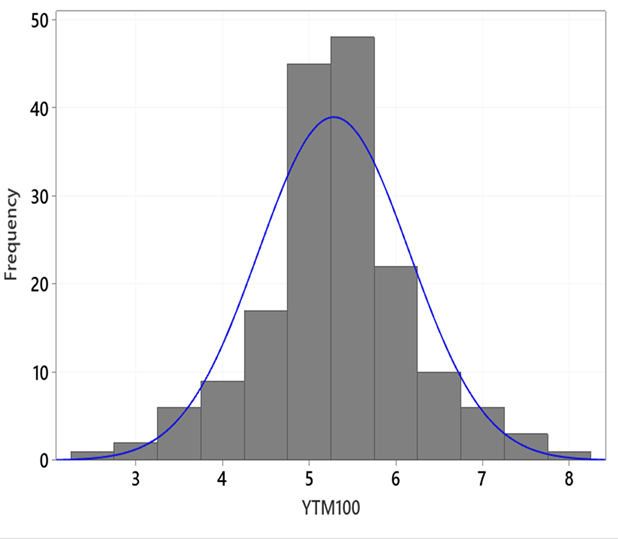


**Supplementary Figure** **2.** Frequency distribution for the BLUPs estimated phenotypic values of agronomic and disease resistance traits of green pea genotypes. PH, plant height; BM, biomass; ERL, earliness; DM, downy mildew; PDL, pod length; PDP, number of pods per plant; PDN, number of pods per plant; PPD, number of peas per pod; YTM100, green pea yield; PSP, percent of small peas.


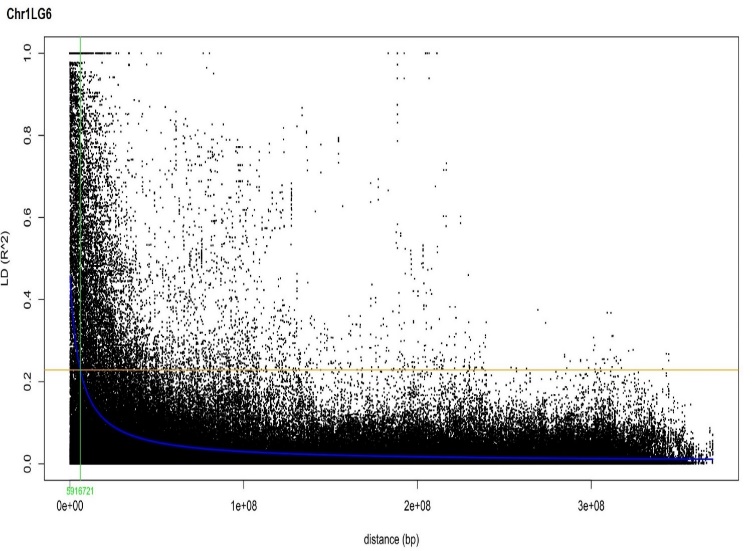

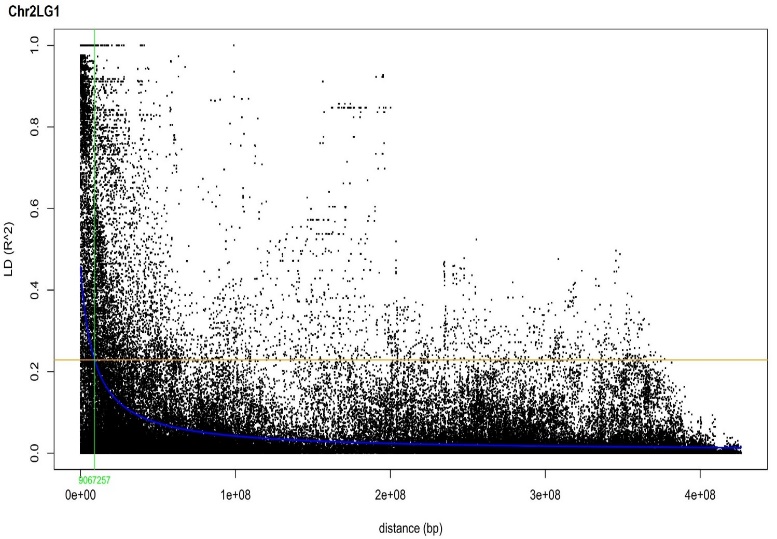

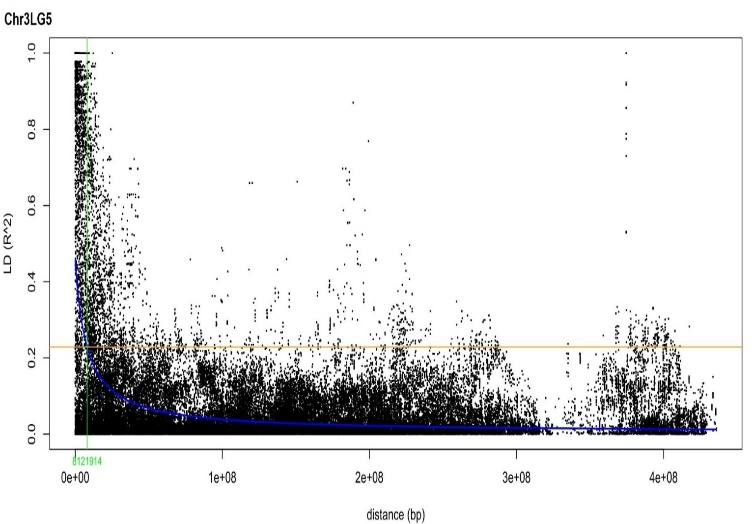

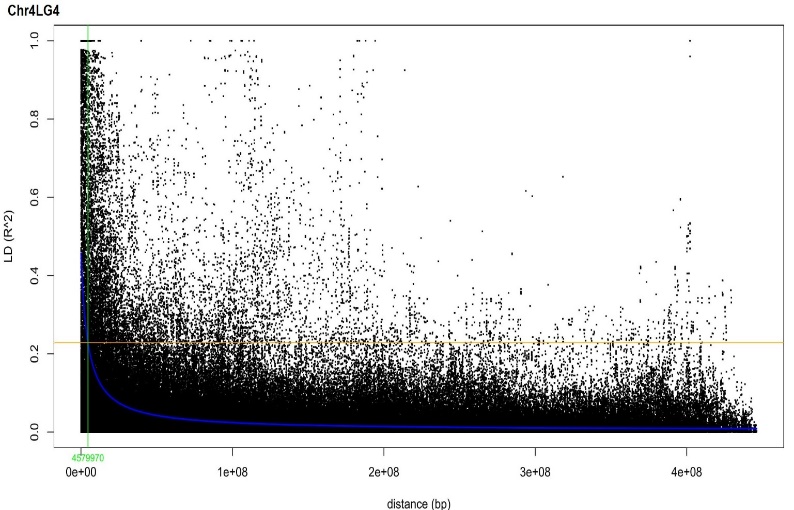

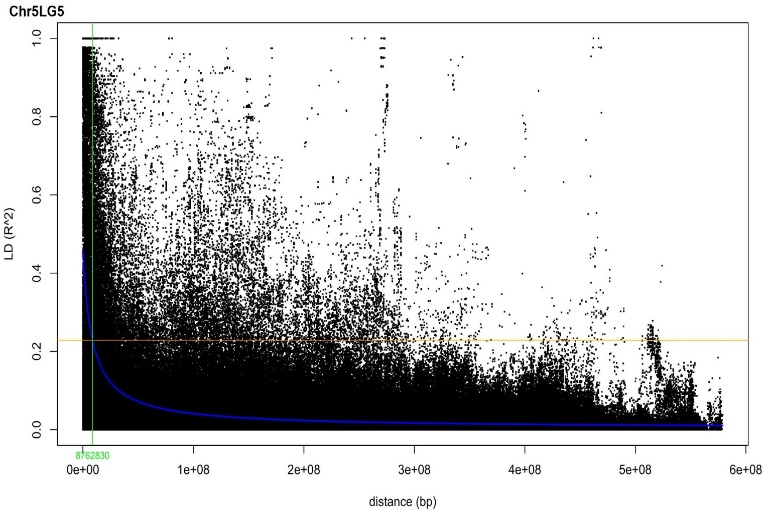

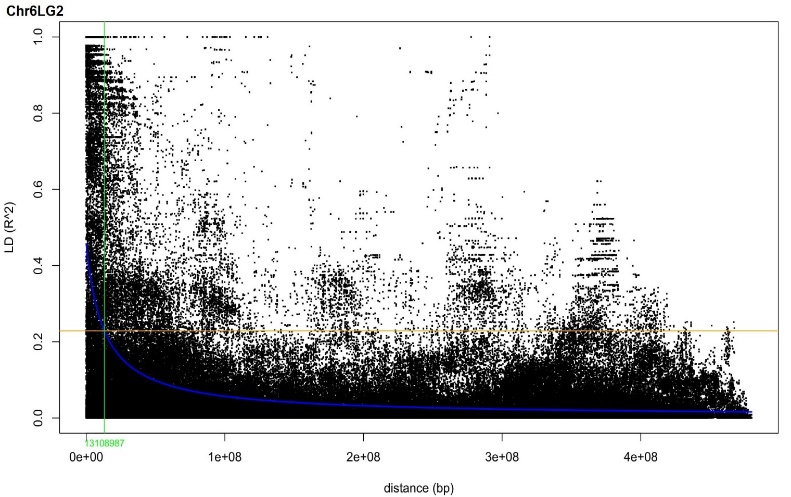

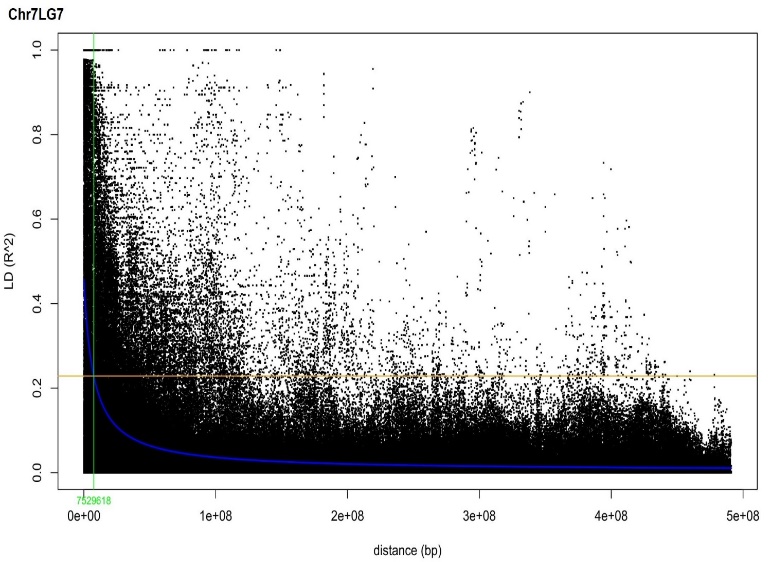

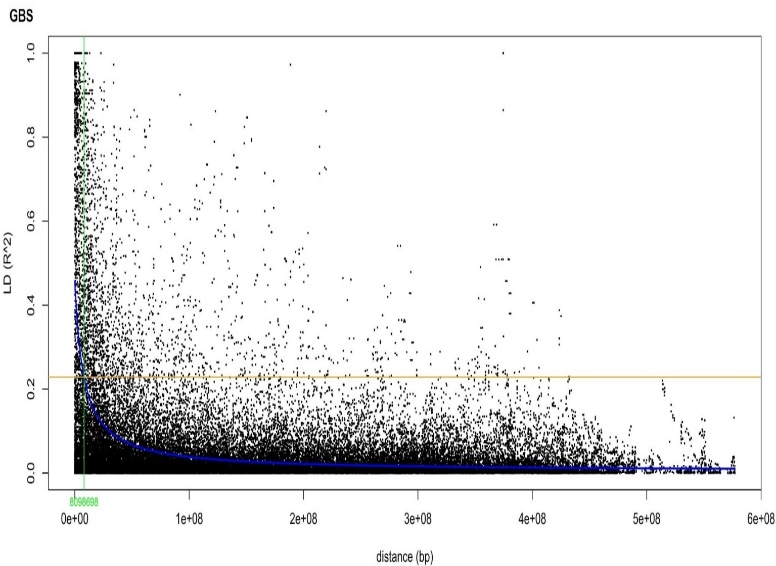

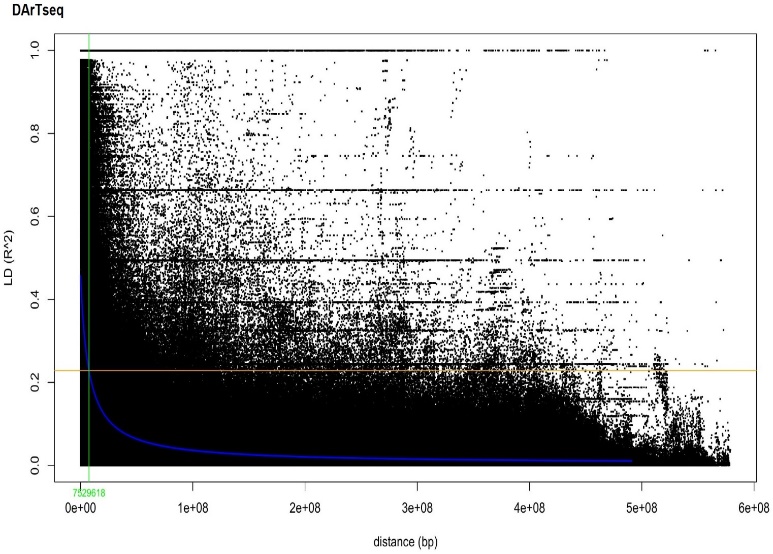


**Supplementary Figure** **3.** Estimated linkage disequilibrium decay for chromosome and whole genome using DArTseq and GBS sequencing methods. The solid blue curve is the smoothing spline regression model fitted to LD decay. The horizontal orange line is the r^2^ value in which half-decay is reached and the green vertical line is the physical distance at which the half-decay intersect with LD decay curve.


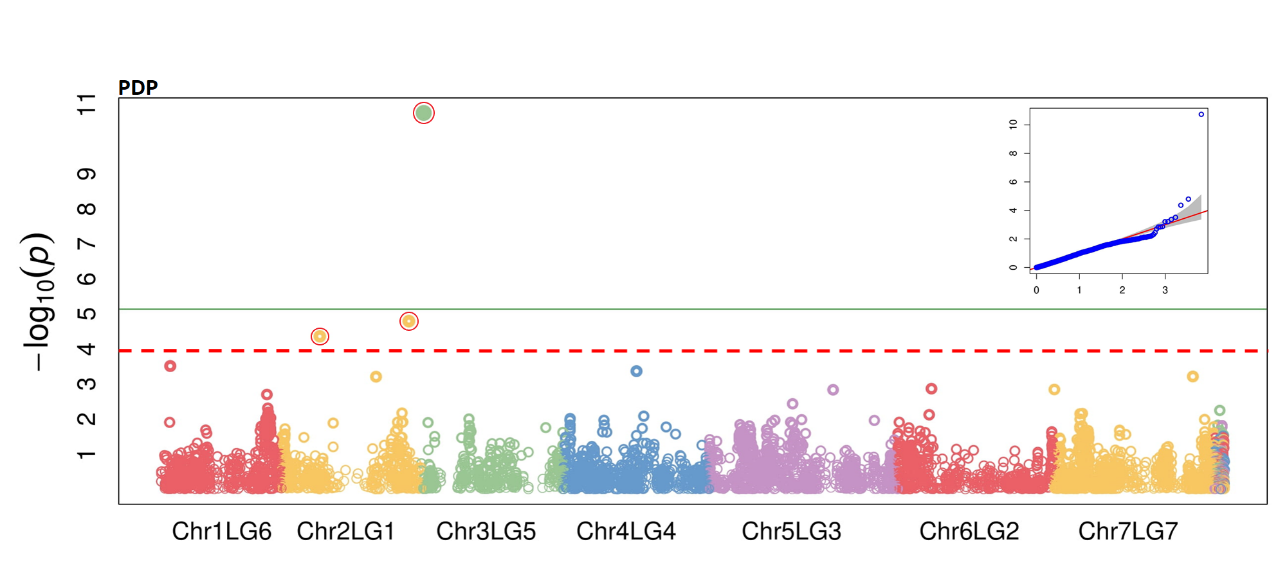

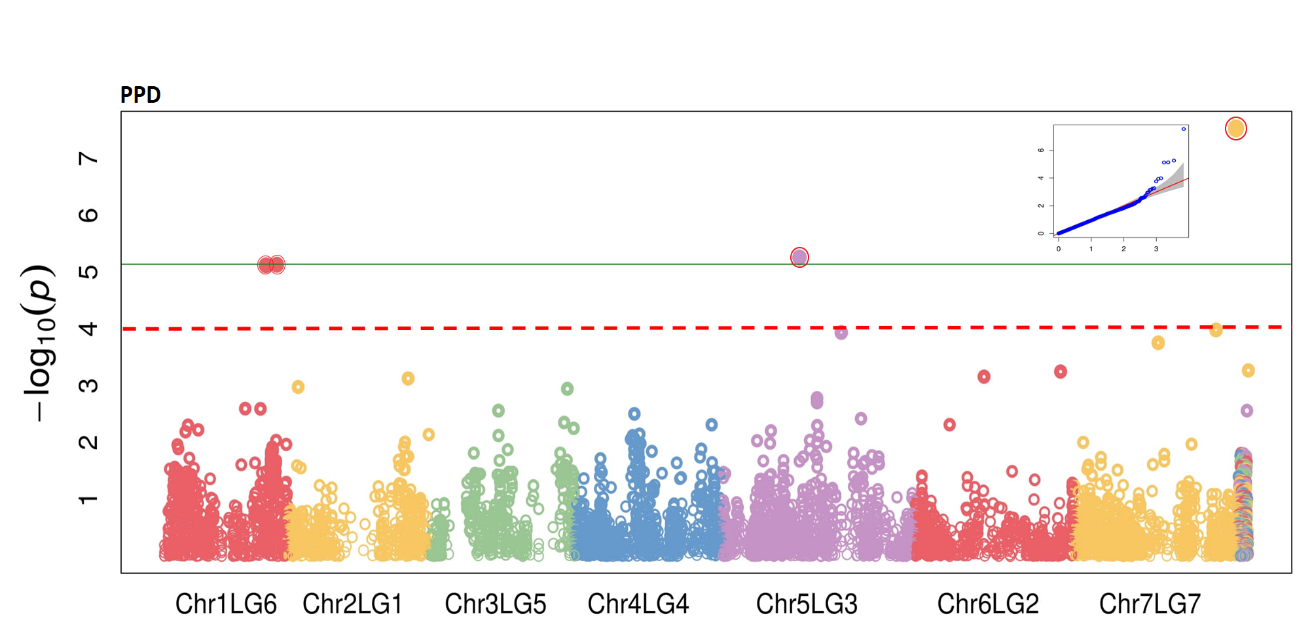

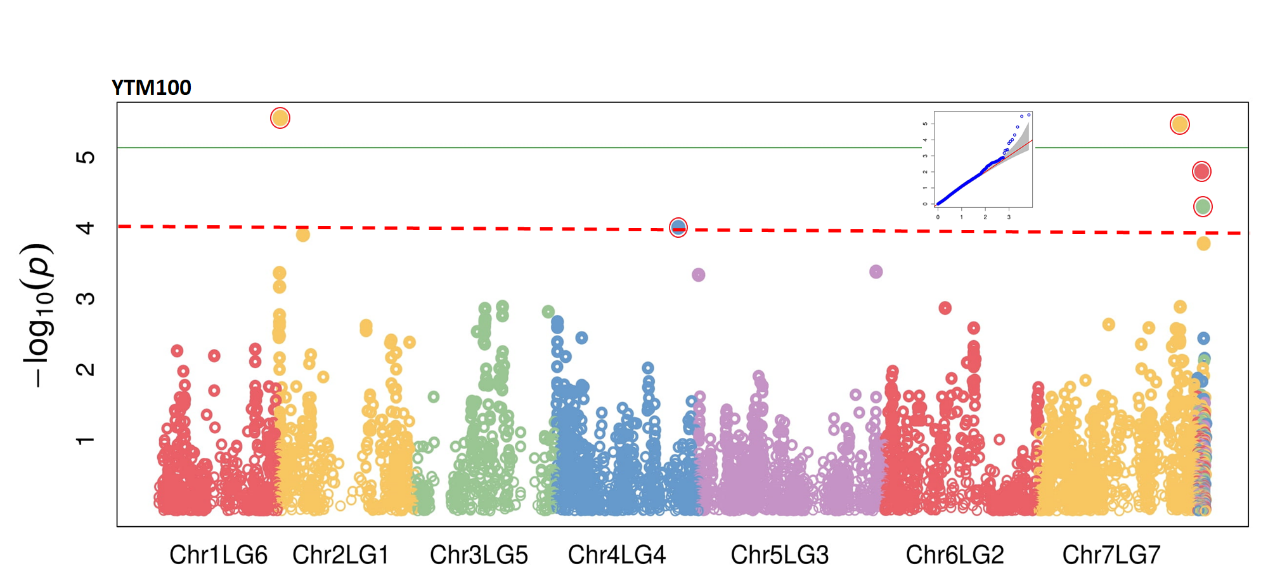


**Supplementary Figure** **4.** Manhattan and quantile-quantile (Q-Q) plots of the GWAS result for agronomic traits of green pea. The horizontal red dash lines and solid green lines indicate the exploratory and Bonforreni thresholds, respectively. PDP, number of pods per plant; PPD, number of peas per pod; YTM100, green pea yield.
